# DNA substrate recognition and processing by the full-length human UPF1 helicase

**DOI:** 10.1093/nar/gkx478

**Published:** 2017-05-24

**Authors:** Saba Dehghani-Tafti, Cyril M. Sanders

**Affiliations:** Department of Oncology & Metabolism, Academic Unit of Molecular oncology, University of Sheffield Medical School, Beech Hill Rd, Sheffield, S10 2RX, UK

## Abstract

UPF1 is a conserved helicase required for nonsense-mediated decay (NMD) regulating mRNA stability in the cytoplasm. Human UPF1 (hUPF1) is also needed for nuclear DNA replication. While loss of NMD is tolerated, loss of hUPF1 induces a DNA damage response and cell cycle arrest. We have analysed nucleic acid (NA) binding and processing by full-length hUPF1. hUPF1 unwinds non-B and B-form DNA and RNA substrates *in vitro*. Unlike many helicases involved in genome stability no hUPF1 binding to DNA structures stabilized by inter-base-pair hydrogen bonding was observed. Alternatively, hUPF1 binds to single-stranded NAs (ssNA) with apparent affinity increasing with substrate length and with no preference for binding RNA or DNA or purine compared to pyrimidine polynucleotides. However, the data show a pronounced nucleobase bias with a preference for binding poly (U) or d(T) while d(A) polymers bind with low affinity. Although the data indicate that hUPF1 must bind a ssNA segments to initiate unwinding they also raise the possibility that hUPF1 has significantly reduced affinity for ssNA structures with stacked bases. Overall, the NA processing activities of hUPF1 are consistent with its function in mRNA regulation and suggest that roles in DNA replication could also be influenced by base sequence.

## INTRODUCTION

Premature termination codons (PTCs) in eukaryotic mRNA transcripts arise frequently due to errors in transcription, RNA processing and from underlying genetic defects (1). Translation of these nonsense transcripts can result in protein products that are detrimental to the cell, e.g. by imposing a dominant negative phenotype, so they are rapidly degraded by a highly conserved nonsense-mediated decay (NMD) pathway. Normal transcripts with a termination codon in close proximity to the poly(A) tail evade NMD, while PTCs are recognized and processed when translation termination occurs distal to a poly(A) site (2). Many proteins are involved in NMD (3) and there is a functional overlap with other related but specialized forms of mRNA regulation, such as Staufen mediated decay (SMD) and histone mRNA decay, which target mRNAs that are otherwise intact (4,5).

Three highly conserved ‘up-frameshift’ proteins, UPF1, UPF2 and UPF3 in human, interact to form a core ‘surveillance complex’ (6) that is recruited to mRNAs destined for NMD. UPF1 is regarded as the master regulator of NMD as it has roles in many steps of the pathway and is also essential for the more discriminate SMD and histone mRNA decay processes (2). *UPF1* was first identified in yeast as a gene involved in the stability of mRNAs with PTCs (7). Subsequent biochemical characterization of yeast Upf1 (8–10) and the human homologue (11–13) demonstrated that the proteins have an RNA binding-dependent ATP*ase* activity and will displace an oligonucleotide from partially single- and double-stranded nucleic acid substrates. ATP*ase* mutants deficient in strand displacement *in vitro* result in loss of UPF1-dependent RNA processing pathways when tested *in vivo*, suggesting that this activity is critical (10,14). UPF2 and UPF3 do not appear to have enzymatic activities but are required for assembly and regulation of a functional surveillance complex (15).

**Figure 1.**
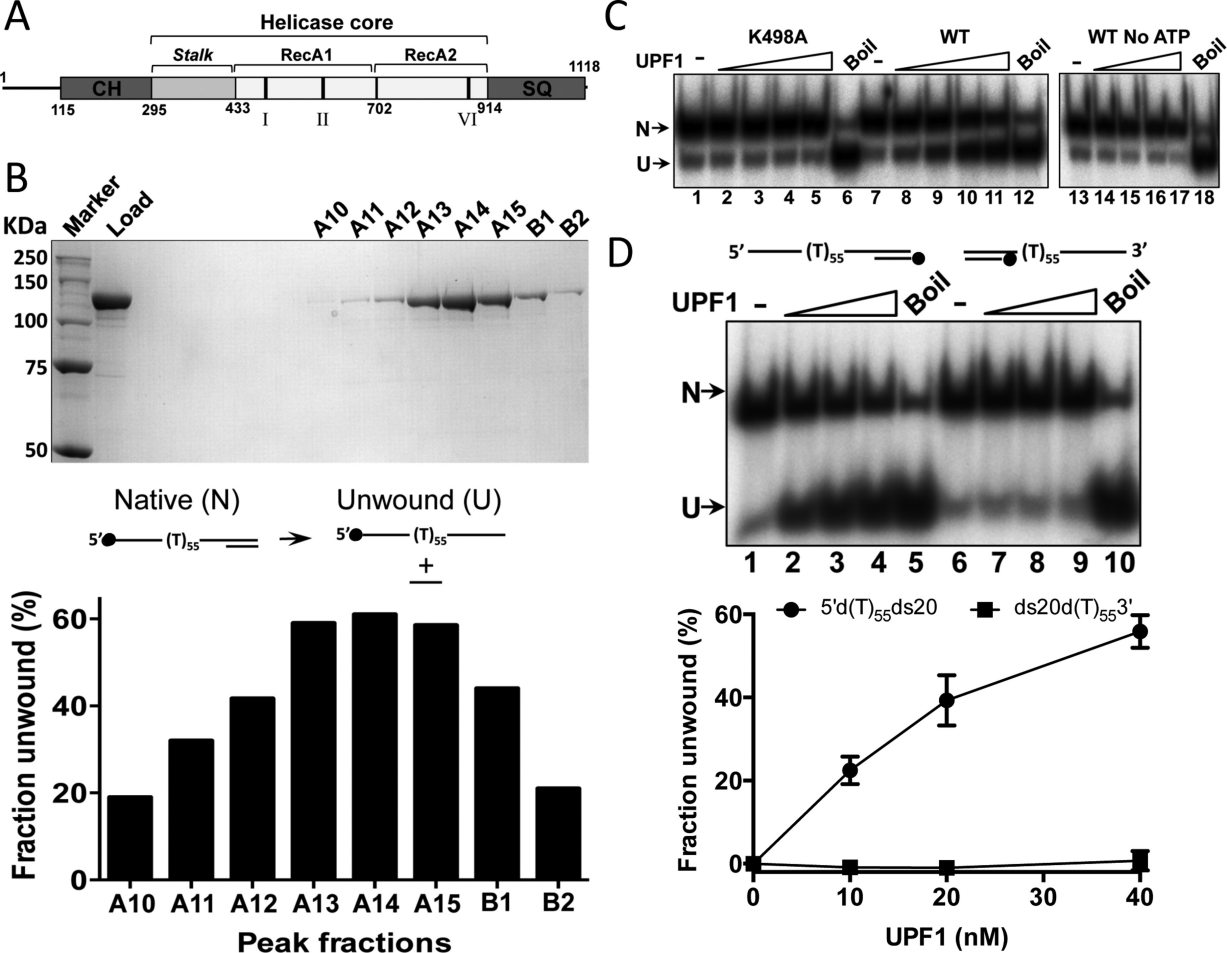
Purification and analysis of full-length human UPF1 (hUPF1). (**A**) Cartoon of the hUPF1 protein (CH, cysteine–histidine rich domain; SQ, serine–glutamine rich domain; I, II and VI, position of conserved ATP*ase* motifs). The substitution K498A was made in motif I (Walker A box) to create the ATP*ase* deficient mutant (13). (**B**) Superdex S200 gel filtration fractions analysed by SDS-PAGE (hUPF1 123 KDa) and their 5΄–3΄ strand unwinding activity (substrate 5΄d(T)_55_ds20). hUPF1 peak elution volume was 12.4 ml, between the BSA (66 KDa) and ferritin (440 KDa) markers. (**C**) Helicase activity (0.2 nM substrate 5΄d(T)_55_ds20, top strand labeled as shown in (B), 4–40 nM hUPF1) was not observed for variant K498A or for wild-type hUPF1 (WT) without ATP. Boil is the thermally denatured substrate. (**D**) hUPF1 displaced a 20 base oligonucleotide from a substrate with a 5΄ poly d(T)_55_ but not a 3΄ poly d(T)_55_ (substrate ds20d(T)_55_3΄) overhang (10–40 nM hUPF1, 0.2 nM substrate, bottom strand labeled), *n* = 3 experimental repeats, mean and standard deviation.

UPF1 is widely recognized as an RNA helicase belonging to helicase superfamily 1 (SF1), the largest helicase superfamily whose members have roles in virtually all aspects of nucleic acid metabolism (16). Helicases use the energy of nucleotide hydrolysis to unwind nucleic acid duplexes and non-B DNA structures such as G-quadruplex (G4) and triplex DNA that form in a sequence dependent manner (17). hUPF1 is a monomeric enzyme (8) and like many eukaryotic helicases it is modular, with a core helicase and auxiliary domains (Figure 1). hUPF1 residues 295–914 contain all the conserved SF1 helicase motifs, and it is also known as the helicase core or the hUPFHD domain (11,13). The hUPFHD structure (13) has two recA-like domains typical of SF1 helicases and a non-conserved ‘stalk’ domain that is essential for NMD and regulates RNA binding affinity in response to ATP. The CH domain (residue 115–295) is a well-conserved cysteine–histidine rich domain. In hUPF1 it binds UPF2 and is an allosteric regulator of RNA binding and helicase activity (15,18,19). The serine–glutamine rich SQ domain is less well conserved and absent in Upf1 from lower eukaryotes. In hUPF1 it also has a negative regulatory effect on the helicase core (20). Finally, N-terminal residues 1–114 are predicted to form a largely unstructured acidic domain.

Early studies concluded that hUPF1 was a cytoplasmic protein (11), consistent with observations that NMD is a cytoplasmic process (21). However, siRNA mediated depletion of hUPF1, but not hUPF2, elicits S-phase arrest and an ATR (Ataxia Telangiectasia and Rad3-related) kinase mediated DNA damage response implicating hUPF1 in genome stability (22). Together with observations that knockout of the *hUPF1* homologue *RENT1* in mice is embryonically lethal (23) while loss of NMD is generally tolerated (24–26), these data suggest an essential role for hUPF1 in DNA replication. hUPF1 is recruited to chromatin during S-phase or when DNA is damaged and a tight association with DNA polymerase δ suggests a direct role at replication forks (22,27). However, along with other NMD factors hUPF1 is also required for telomere replication (28,29). Both of these replication functions are modulated by ATR-dependent hUPF1 phosphorylation.

The role of hUPF1 in mRNA regulation is well studied but its interactions with DNA are not. Biochemical studies have been restricted due to difficulties in producing intact hUPF1 and have focused mainly on the analysis of truncated species (13,15,18–20). We have purified full-length hUPF1 expressed in *Escherichia coli* and have analysed its nucleic acid (NA) binding and unwinding properties. We show that hUPF1 unwinds NA duplexes as well as G quadruplex (G4) and triplex DNA structures implicated in genetic instability, but unlike many of the helicases involved in genome stability we were unable to detect any specific binding of hUPF1 to DNA secondary structures that are stabilized by inter-base-pair hydrogen bonding. However, hUPF1 binds avidly to ssNAs and the apparent affinity is oligonucleotide length dependent. Surprisingly, the ssNA binding activity is heavily influenced by the nucleobases. Although there is no bias in binding purine compared to pyrimidine polynucleotides or RNA compared to DNA, homopolymeric ribo- and deoxyribonuleotides that may form single-stranded helices by base stacking are bound with low affinity. We discuss these new findings in relation to the known cellular roles of the enzyme.

## MATERIALS AND METHODS

### Expression and purification of hUPF1

The full-length hUPF1 ORF and a Walker A ATP*ase* motif mutant (K498A) were cloned in pET11c with N-terminal glutathione S-transferase and C-terminal poly histidine affinity tags. The C-terminal tag consisted of a small linker (ASGL) followed by the TEV cleavage sequence (ENLYFQS) and six histidine residues. The N-terminal GST tag was followed by a thrombin cleavage site. Tagged UPF was expressed in *E. coli* BL21(DE3) for 6 h at 16°C after induction at OD_600_ ∼0.8. All purification steps were at 4°C. Cells were re-suspended in lysis buffer (50 mM Tris–HCl pH 7.5, 0.1 M NaCl, 10 mM EDTA, 10% v/v glycerol, 10 mM DTT and 1 mM PMSF) at the ratio of 1ml per 1.5 g of cells and incubated with lysozyme (1 mg/ml) for 30 min. After addition of the same volume of lysis buffer/1.9 M NaCl cells were lysed by sonication and the lysate cleared (40 000 × *g*, 30 min). Nucleic acids were removed by precipitation with polyethylenimine P (0.5% w/v) and proteins precipitated with ammonium sulphate (50% saturation) before GST affinity chromatography. Eluted protein was digested with thrombin and concentrated by binding to and step elution from a 1 ml Ni-sepharose ‘His-Trap’ column (GE Healthcare) before application to a Superdex 200 (XK16/100, GE Healthcare) column (25 mM Tris–HCl pH 7.5, 0.2 M NaCl, 10% v/v glycerol, 5 mM DTT and 1 mM PMSF. The low molecular weight hUPF1 fraction was then re-applied to a His-Trap column and eluted in a gradient from 20 to 250 mM imidazole (50 mM Tris–HCl pH 7.5, 0.5 M NaCl, 10% v/v glycerol, 2.5 mM DTT). hUPF1 peak fractions were dialysed against >100 volumes of 25 mM Tris–HCl pH 7.5, 0.2 M NaCl, 10% v/v glycerol, 2.5 mM DTT and digested with TEV protease to remove the His-Tag. The protein was re-applied to the His-Trap column and the flow through was concentrated and re-applied to a high-resolution Superdex 200 (10/300) gel filtration column. Peak fractions were concentrated to ∼1 mg/ml and stored at –80°C. Protein concentration was determined by BioRad assay using bovine serum albumin (BSA) as a standard.

### Helicase substrates

Oligonucleotides were purchased from Sigma Aldrich. The sequence and composition of all substrates used are described in detail in Supplementary Figure S1. Oligonucleotides were end-labeled with ^32^P using polynucleotide kinase and [γ-^32^P]ATP (6000 Ci/mmol) and the final substrates resolved on 8% (19:1) poly-acrylamide gels (1 × TBE running buffer, 89 mM Tris-borate, 2 mM EDTA), before recovery by the crush and soak elution method.

All the linear partially single- and double-stranded NA substrates contained a sequence that would anneal to a 20 bp complementary oligonucleotide 5΄-dGGGTACCGAGCTCGAATTCG. Generation of the tetramolecular G4 DNA substrates have been described previously (30). For the RNA:DNA hybrid substrates the RNA component (Supplementary Figure S1) was generated by run-off transcription from a linearised plasmid substrate using T7 RNA polymerase (31). All RNA transcripts generated as such begin with three G residues derived from the T7 RNA promoter sequence.

The triplex forming DNA was based on the sequence 5΄-GGGGAGGGGACGGTGAAG from the human rhodopsin gene (32) imbedded in a 93 bp duplex (Supplementary Figure S1). The template DNA sequence was cloned between the Sal I and Sma I sites of pUC19 and amplified by primer extension using primers TripL (5΄-dACGTTCTAGAGCGCGCGCCACCCAGC) and TripR (5΄-dTGCATCTAGATCTAAGCCGACTGGCG), one of which was end labelled with ^32^P, as described above. The dsDNA product was purified using a QiaQuick column (Qiagen) before annealing with a 5-fold excess of strand three of the triplex forming DNA (∼2.5 pmol/μl dsDNA, 10 mM Tris-HCl pH 7.5, 10 mM MgCl_2_, 10% w/v glycerol; reactions incubated in a boiling water bath for 5 min before slowly cooling to 4°C). Products were purified on an 8% polyacrylamide gel (89 mM Tris-borate, 10 mM MgCl_2_, pH 8.3) and eluted in triplex annealing buffer. Triplex formation was confirmed by methylation protection using dimethyl sulphate (DMS) (31) and analysis of the products on a 10% urea-PAGE sequencing gel.

### Helicase assays

Strand displacement assays (0.2 nM radiolabelled substrate) were performed in 25 mM HEPES–NaOH pH 7.2, 75 mM NaCl, 2 mM DTT, 5 mM ATP, 5 mM MgCl_2_, 0.1 mg/ml BSA at 37°C for 30 min with the indicated concentrations of hUPF1. For the analysis of triplex unwinding the MgCl_2_ concentration was increased to 10 mM. Reactions were terminated by the addition of 0.25 vol. stop buffer (60% v/v glycerol, 0.5 mg/ml bromophenol blue, 0.25% (w/v) SDS, 100 mM EDTA pH 8.0, 1 μM T55 ssDNA and 10 ng/ul pUC19 plasmid DNA) and analyzed on 8% (19:1) polyacrylamide gels with 0.05% w/v SDS and 1 × TBE/0.05% (w/v) SDS running buffer. The stop buffer for analysis of triplex unwinding contained no EDTA, and the gel and electrophoresis buffers contained 10 mM MgCl_2_ and no EDTA. The gel and TBE running buffer for analyzing G4 DNA unwinding contained 0.1% (w/v) sarkosyl and 50 mM KCl. The dried gels were visualized and quantified by phosphorimager. Enzymatic strand displacement was calculated after subtraction of the fraction of non-enzymatically dissociated substrate observed in control reactions. All data presented in the graphs are derived from a minimum of three repeats against an independent dilution series of protein and show the mean with the standard deviation indicated with error bars.

### DNA binding assays

DNA substrates were end-labeled (^32^P) and purified as described above. The 35 base single-stranded DNA and RNA oligonucledotides were purified by denaturing PAGE and quantified by UV spectroscopy using the calculated molar extinction coefficients. The sequence of the DNA_35_ substrate was 5΄-dCACAAGCAACCAATCGGTTCGACACTCATACTGGC and the RNA_35_ substrate 5΄-CACAAGCAACCAAUCGGUUCGACACUCAUACUGGC.

DNA binding reactions were performed in 25 mM HEPES–NaOH pH 7.2, 135 mM NaCl, 2 mM DTT, 1 mg/ml acetylated BSA (Promega) and 0.1% NP40, with and without nucleotide cofactors and MgCl_2_ as indicated, at 20°C for 20 min with the indicated concentrations of hUPF1. Complexes were resolved on 8% polyacrylamide gels (29:1, 0.25 × TBE buffer) and dried gels were visualized and quantified by phosphorimager (electrophoretic mobility shift assay, EMSA). All data presented in the graphs are derived from a minimum of three independent repeats and shows the mean and standard deviation delimited by the error bars.

### Microscale thermophoresis (MST)

DNA substrates d(A)_35_, d(C)_35_, d(T)_35_, d(T)_25_, d(T)_15_ and the heteropolymer DNA_35_, as described above, were synthesized 5΄ end-labeled with Alexa 647 dye and HPLC purified (Sigma Aldrich). The binding reaction buffer conditions for MST were exactly the same as for the gel-shift (EMSA) reactions. The concentration of labeled DNA was set at 20 nM and hUPF1 was titrated from 0.0381–1250 nM. Samples were loaded into Monolith NT.115 MST standard treated capillaries (NanoTemper Technologies) and MST measured after 20 min incubation at 22°C using a Monolith NT.115 and MO.Control software (Version 1.44, LED/excitation power setting 20%, MST power setting 40%). Data were analysed using the MO.Affinity Analysis software (version 2.2.5, NanoTemper Technologies) at an MST-on time of 10 s. Each substrate was analysed in triplicate against three independent protein dilution series. SDS-denaturation tests were performed to rule out non-specific absorption and confirm that fluorescent changes were induced by hUPF1 binding.

## RESULTS

### Production of full-length recombinant hUPF1

Purification of recombinant full-length hUPF1 was achieved with N- and C-terminal affinity tags (GST and (His)_6_ respectively) after expression in *E. coli* using a pET vector. The final product, free from affinity tags removed by site-specific protease digestion, eluted from a high-resolution gel filtration column as a single peak indicative of mono-dispersed hUPF1 (Figure 1B). Approximately 1 mg of purified hUPF1 protein was obtained from ∼300 g wet-weight of *E. coli* cells (∼3.3 μg hUPF1 per gram of cells). The solubility of the protein in the final buffer appeared to be limited to ∼1 mg ml^−1^ as attempts to concentrate it further by membrane ultrafiltration resulted in no further increase in the protein concentration.

Although hUPF1 is recognized principally as a 5΄–3΄ RNA helicase, where the enzyme engages with a 5΄ single-stranded NA tail and translocates upon it during strand displacement (19), we used a partially single- and double-stranded DNA test substrate with a 55 base 5΄ poly d(T) tail and 20 bp dsDNA to monitor helicase activity of the peak fractions. As shown in Figure 1B, maximum strand displacement activity corresponded with the peak protein fraction from the gel filtration column (A14), which migrated in SDS-PAGE with a molecular weight consistent with full-length hUPF1 (123 kDa). A variant hUPF1 protein with an amino acid substitution K498A in ATP*ase* motif I (Walker A box, Figure 1A) was also purified exactly as the wild-type. The K498A substitution has been shown previously to abolish the ATP*ase* activity of the hUPF1 helicase core (13). K498A hUPF1 had no strand displacement activity (Figure 1C, lanes 2–5 compared to 8–11) and no helicase activity was detected for the wild-type enzyme in the absence of ATP (lanes 14–17). Furthermore, recombinant hUPF1 could effectively displace a 20 base oligonucleotide from a duplex with a 55 base 5΄poly d(T) tail, but not one with a 55 base 3΄poly d(T) tail (Figure 1D). In general for the simple test substrate 5΄d(T)_55_ds20 we did not observe any significant increase in strand displacement activity above ∼60% when assayed at hUPF1 concentrations > 40 nM. One possible explanation for this could be the combined relatively low solubility of the protein and its tendency to multimerise in the presence of ssNAs (see below).

### RNA and DNA helicase activity of hUPF1

Optimal conditions for hUPF1 nucleic acid unwinding (see materials and methods) were determined using the DNA substrate with a 55 base 5΄ poly d(T) tail and a 20bp dsDNA segment (Supplementary Figure S2). Further analysis demonstrated detectable unwinding with a 5΄ ssDNA tail length of 15 d(T) but not 5 d(T) residues and increasing unwinding with tail lengths up to 45 d(T) residues whereupon further increases in tail length had a minimal effect (Supplementary Figure S2). We therefore adopted the substrate with a 5΄ poly d(T)_55_ tail and 20 bp dsDNA (5΄d(T)_55_ds20) as a standard for comparison with all other substrates in helicase assays. Modification of substrate 5΄d(T)_55_ds20 to a fork-like substrate with 5΄d(T)_55_ and 3΄d(C)_30_ tails did not alter the efficiency of unwinding of the 20 bp duplex (Supplementary Figure S3).

**Figure 2.**
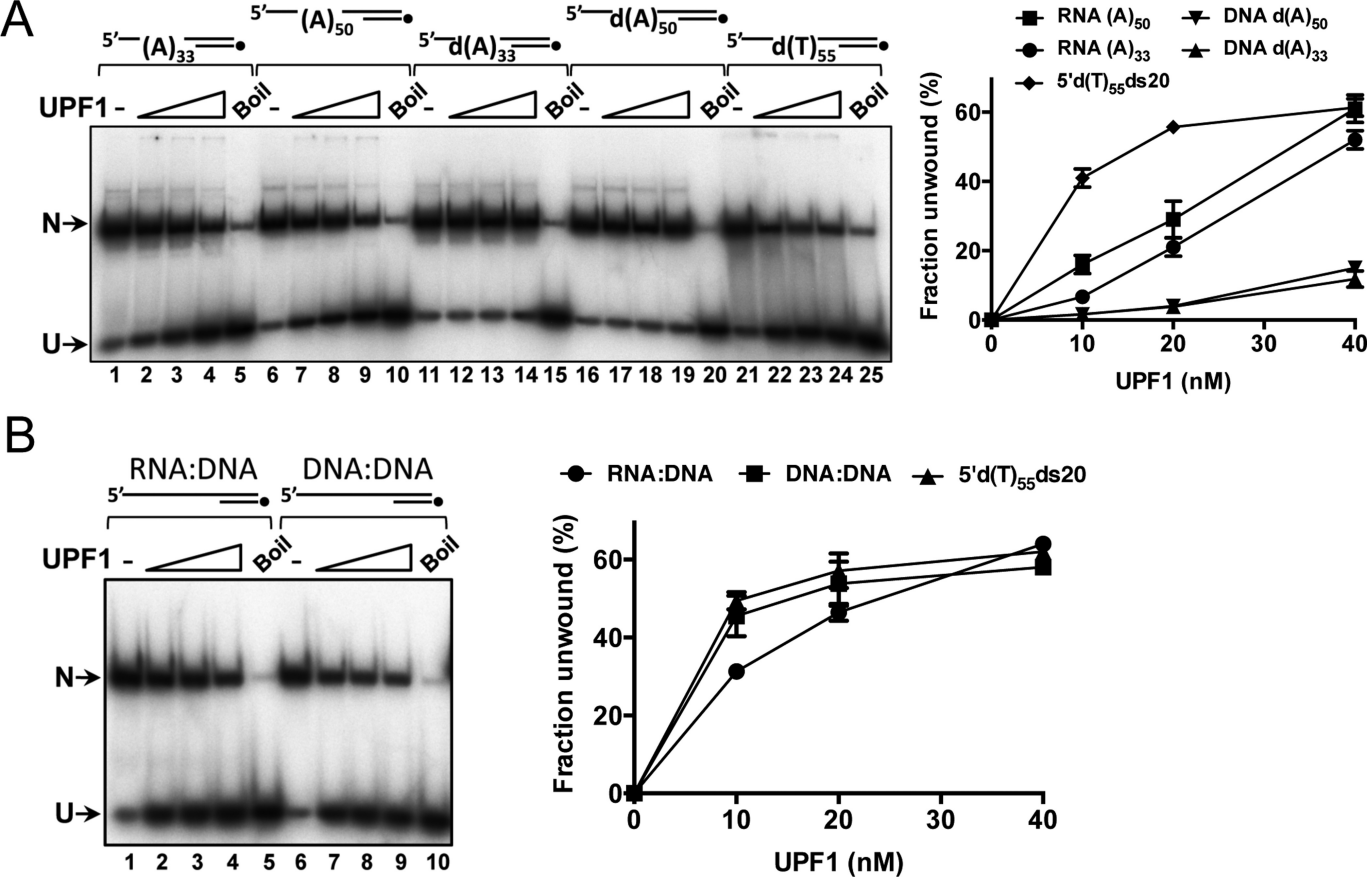
Unwinding of duplex DNA and RNA:DNA hybrids. (**A**) Tracking strands were 50 or 33 base 5΄ poly (A) or d(A) extension, preceded by 3 G residues (see note in materials and methods and Supplementary Figure S1). For simplicity these substrates are referred to as RNA (A)_50_, RNA (A)_33_, DNA d(A)_50_ and DNA d(A)_33_. Compared to 5΄d(T)_55_ds20, substrates with 5΄ poly d(A) tails were poor helicase substrates (∼20 fold less efficient at 10 nM UPF1 for substrates with comparable tail lengths) and RNA:DNA hybrids showed intermediate levels of unwinding (∼40% unwinding efficiency for substrates with similar tail lengths). (**B**) Substrates (20 bp duplex as in (A)) with 5΄ 55 base extension of the corresponding RNA or DNA heteropolymer sequence compared to substrate 5΄d(T)_55_ds20 in helicase assays. All reactions contained 0.2 nM substrate and 10, 20 or 40 nM hUPF1, *n = 3* experimental repeats, mean and standard deviation.

Since the unwinding of the DNA substrate 5΄d(T)_55_ds20 appeared robust compared with the reported activity of truncated hUPF1 species (15,19,20), RNA and DNA helicase activities were analysed further. We measured the ability of hUPF1 to displace a 20 base ^32^P end-labeled oligonucleotide from complementary DNA or RNA oligonucleotides with 5΄ single-stranded extensions (Figure 2). In each case the sequence of the hybridised 20 base oligonucleotide was identical. Surprisingly, DNA substrates with extended 5΄ poly d(A) tails (DNA d(A)_50_ and d(A)_33_, Figure 2A) were poor substrates for hUPF1 catalyzed dsDNA unwinding (Figure 2A, lanes 11–20 and graphed data to the right) compared to 5΄d(T)_55_ds20. However, strand displacement from the RNA oligonucleotide with 30 or 50 base 5΄ poly (A) tails (lanes 1–10) was greater than the corresponding d(A) tailed substrates, but less efficient than the reference 5΄d(T)_55_ds20 substrate.

Employing all nucleobases, we also designed 75 base oligonucleotides with the same ribo- or corresponding deoxyribonuclotide sequence for annealing to the 20 base DNA oligonucleotide, generating helicase substrates with 55 base 5΄ tails (Supplementary Figure S1). The online web server mfold (33) was used to minimize secondary structure in the single-stranded nucleic acid segments. In hUPF1 helicase assays the extent of unwinding of the DNA substrate and reference 5΄d(T)_55_ds20 as a function of protein concentration were comparable (Figure 2B), while the RNA:DNA hybrid was less efficiently unwound at the lower protein concentrations (∼30% at 10 nM hUPF1). Together, the data for unwinding simple partially single- and double-stranded nucleic acid substrate show that hUPF1 can engage and translocate effectively on DNA as well as RNA to catalyse strand displacement. However, the substrates employed with mononucleotide repeat 5΄ single-stranded tails indicate that the unwinding reaction is sensitive to the nucleotide composition of this segment of the substrate.

### hUPF1 unwinds triplex DNA

**Figure 3.**
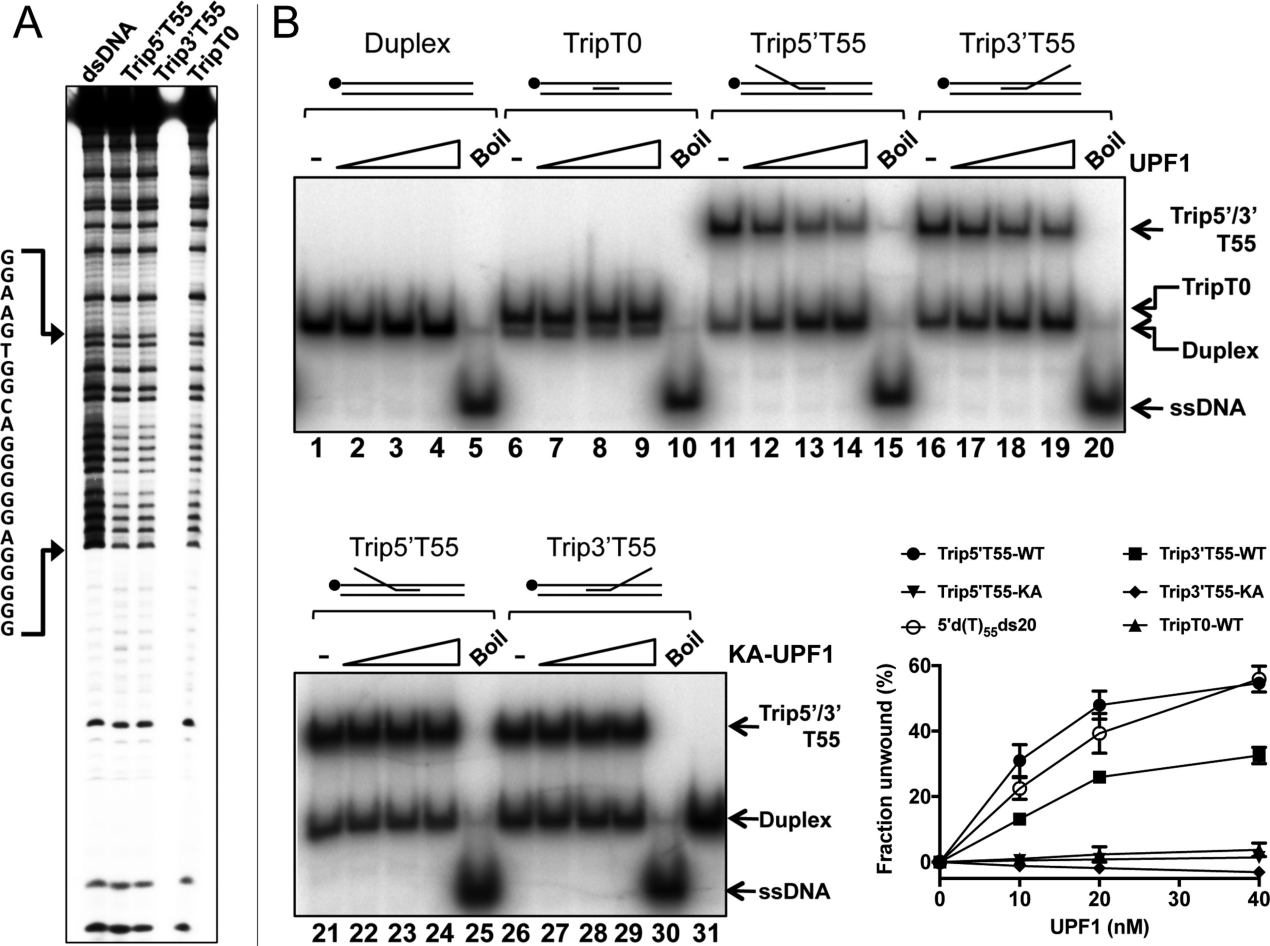
hUPF1 unwinds triplex DNA. (**A**) Methylation protection of the triplex substrates without (TripT0) or with d(T)_55_ 3΄ or 5΄ extensions to the triplex forming oligonucleotide (Trip3΄T55 and Trip5΄T55). The top strand of the duplex/partially triplex sequence was ^32^P-end labeled. (**B**) Helicase assays, 10–40 nM hUPF1 or variant K498A (KA-UPF1), 0.2 nM ^32^P-end labeled substrate (5΄ end, top strand of parent duplex). Duplex DNA and substrate TripT0 (no ssDNA component) were not unwound. Trip5΄T55 was resolved with an efficiency approaching that of substrate 5΄d(T)_55_ds20 (run in parallel but not shown in (B)). Substrate Trip3΄T55 was also resolved by hUPF1 at ∼70% efficiency compared to substrate Trip5΄T55. All substrates were analysed in parallel, *n* = 3 experimental repeats, mean and standard deviation shown in the graph.

Intermolecular triplex substrates were generated by annealing oligonucleotides with a 21 base triplex forming sequence (32) without (substrate TripT0) or with 55 base 5΄ or 3΄ poly d(T)_55_ extensions (substrates Trip5΄T55 and Trip3΄T55) within a 96 base pair sequence (Supplementary Figure S1). In triplex DNA the N7 position of purines is protected from DMS methylation by Hoogsteen base pairing, while in dsDNA it is reactive resulting in modified bases that can be cleaved with piperidine. In the sequencing gel shown in Figure 3A all triplex substrates display significantly reduced strand cleavage over the G-rich triplex forming motif relative to control dsDNA (lane 1), confirming that the majority of the substrate is triplex DNA. The triplex substrates however displayed a lower intrinsic stability during experimental manipulation compared to duplex substrates. As with all helicase assays, enzymatic strand displacement was calculated after subtraction of the fraction of non-enzymatically dissociated substrate observed in control reactions.

Increasing concentrations of hUPF1 did not unwind duplex DNA (Figure 3B, lanes 1–4) nor could the enzyme efficiently displace the 21 base triplex forming oligonucleotide from the partially duplex and triplex substrate TripT0 (Figure 3B lanes 6–9 and graphed data). Surprisingly however, hUPF1 could effectively displace the triplex forming oligonucleotide from substrate Trip5΄T55 and Trip3΄T55, although the extents of displacement observed with the 3΄d(T)_55_ tail were approximately 60% of those observed with the 5΄-d(T)_55_ extension (Figure 3B lanes 11–14 compared to 16–19 and the graph shown). The K498A variant of hUPF1 did not catalyse strand displacement from triplex substrates with 5΄ or 3΄ extensions (Figure 3B, lanes 21–24 and 26–29), nor was significant strand displacement observed in the absence of ATP (Supplementary Figure S4). Furthermore, we observed a modest decrease in the proportion (<4%) of non-enzymatically dissociated substrate in reaction containing substrate Trip3΄T55 and hUPF1-K498A.

### hUPF1 unwinds G quadruplex DNA

**Figure 4.**
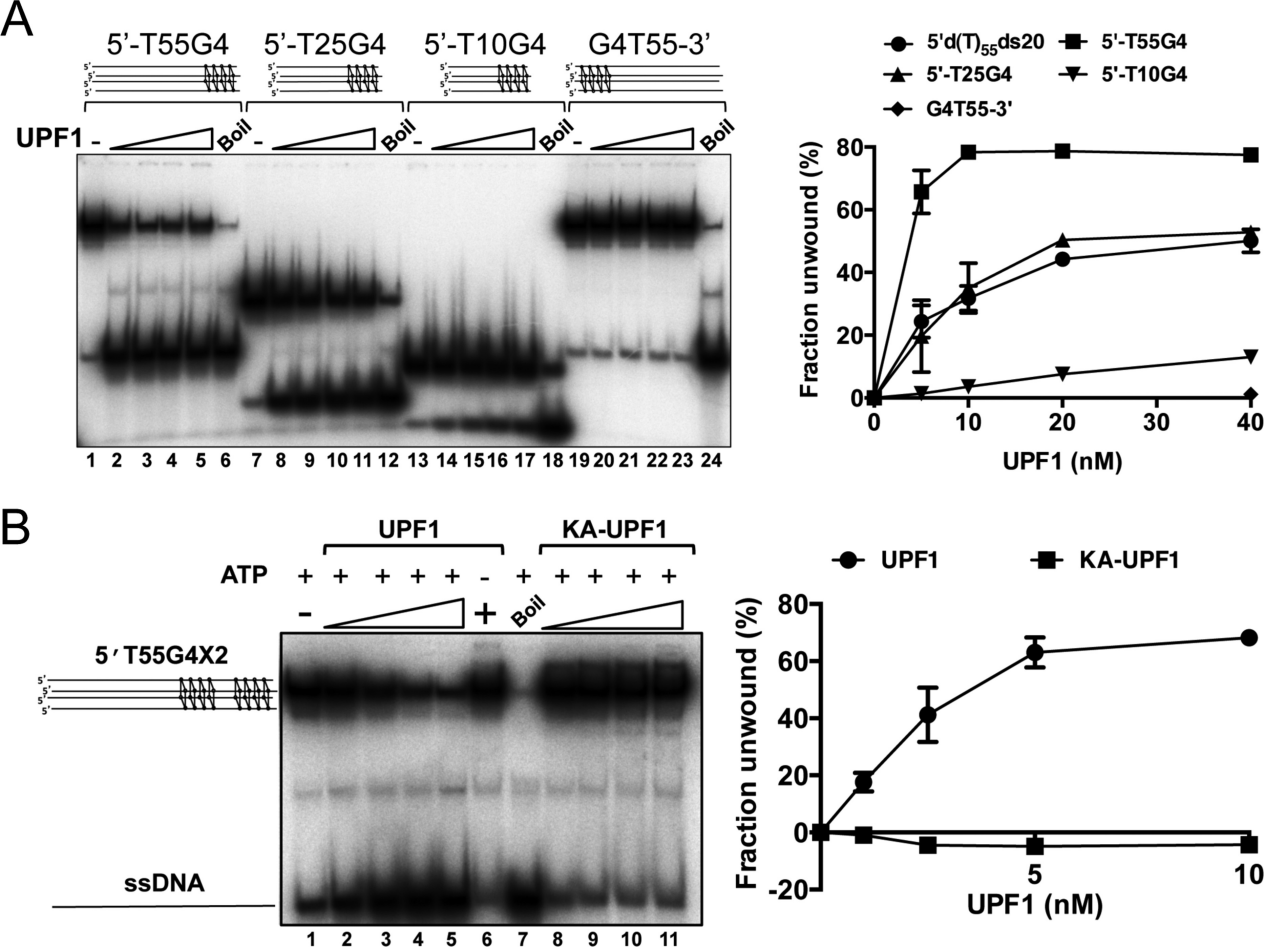
hUPF1 unwinds G quadruplex DNA. (**A**) Synthetic tetramolecular G4 substrates with four guanine quartets and 5΄-d(T) extensions (55, 25 and 10 bases; lanes 1–18) were resolved by hUPF1 (5–40 nM, 0.2 nM substrate), but not a substrate with a 3΄-d(T)_55_ extension (lanes 19–23). The unwinding efficiency was proportional to 5΄-d(T) tail length as shown in the graph to the right (includes data for 5΄d(T)_55_ds20 analyzed in parallel). (**B**) hUPF1 resolves tetramolecular G4 substrates with two sets of four guanine quartets (1–10 nM UPF1, 0.2 nM substrate, lanes 2–5), but not in the absence of ATP (lane 6, 10 nM UPF1). KA-UPF1 failed to unwind G4 DNA, lanes 8–11. *n* = 3 experimental repeats, mean and standard deviation shown.

Synthetic parallel tetramolecular G4 DNA substrates with four or eight G tetrads were generated with oligonucleotides containing the sequence 5΄-dGGGG or 5΄-dGGGGTTTTGGGG and 5΄ or 3΄ poly d(T)_*n*_ extensions. Tetramolecular G4 substrates with 4 G tetrads and 5΄-d(T) extensions of 55, 25 or 10 residues were effectively resolved to a single-stranded product by hUPF1 (Figure 4A). Furthermore, the efficiency of resolution was dependent on the 5΄ ssDNA tail length, closely paralleling the dependence on 5΄ tail length observed for the unwinding of simple partially single- and double-stranded test substrates (Supplementary Figure S2). Compared to all the other substrate, the G4 substrate with the 55 base 5΄-d(T) extensions was efficiently unwound at the lowest protein concentration tested, indicating that hUPF1 may bind to more than one 5΄ tail and cooperate in unwinding. However, as noted above for the partially single- and double-stranded substrates, we also observed inhibition of unwinding at the higher protein concentrations tested (lanes 2–5). The G4 substrate with a d(T)_55_ 3΄ extension was not unwound by hUPF1 (Figure 4A, lanes 19–23 and graph to the right).

A tetramolecular G4 substrate with eight G tetrads and a 5΄-d(T)_55_ tail was also tested in the unwinding assay (Figure 4B). This substrate was also effectively resolved by wild-type hUPF1 in the presence of ATP (lanes 2–5) but not in its absence (lane 6) or by the hUPF1 variant K498A in the presence of ATP (lanes 8–11). However, quantification of the substrate and product (lanes 8–11) consistently revealed a small (∼5% max.) decrease in the proportion of single-stranded product compared to native substrate in each reaction, relative to the enzyme-independent dissociation of substrate (lane 1). A similar observation was made when the triplex substrates Trip5΄T55 (5΄-d(T)_55_ ssDNA extension) but not Trip3΄T55 was analysed in the absence of ATP (Supplementary Figure S4). We have not observed a strand re-annealing activity for hUPF1 using complementary duplex or G4 forming test substrates (data not shown). A possible explanation for this observation is that hUPF1 binding to ssDNA (see below) stabilizes the G4 DNA substrate. The stabilization effect also appears to reflect the unwinding polarity of the enzyme.

### Binding of hUPF1 to poly d(T) oligonucleotides

**Figure 5.**
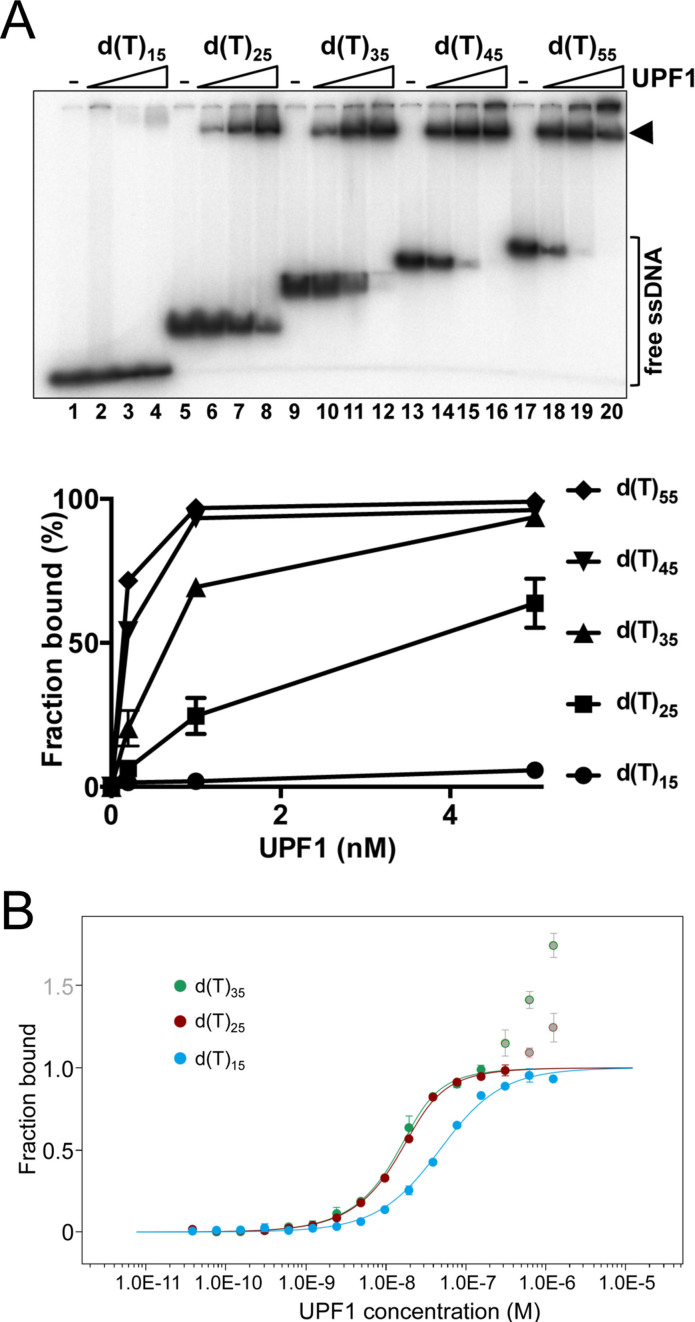
Length-dependent binding of hUPF1 to oligo d(T) substrates. (**A**) EMSA analysis (0.1 nM substrate, 0.2–5 nM hUPF1). hUPF1 formed a single discrete complex with increasing apparent affinity on d(T) oligonucleotide up to 35 bases in length. The bound fraction was taken as all shifted species. (**B**) hUPF1 (0.0381–1250 nM) binding to Alexa 647-labeled d(T)_35_, d(T)_25_ and d(T)_15_ substrates analysed by MST. Biphasic curves were obtained with ligands d(T)_25_ and d(T)_35_. The second event was interpreted as protein multimerization as observed in gel-shift analysis. To determine binding constants data for d(T)_35_ were analyzed up to 156 nM hUPF1 and for d(T)_25_ up to 313 nM hUPF1. Discarded values are indicated as grey circles with colored rims. Apparent *K*_d_ values of 4.05 ± 0.58 × 10^−9^, 5.98 ± 0.51 × 10^−9^ and 3.94 ± 0.36 × 10^−8^ M were determined for d(T)_35_, d(T)_25_ and d(T)_15_ respectively. EMSA and MST, *n* = 3 experimental repeats, mean and standard deviation.

**Figure 6.**
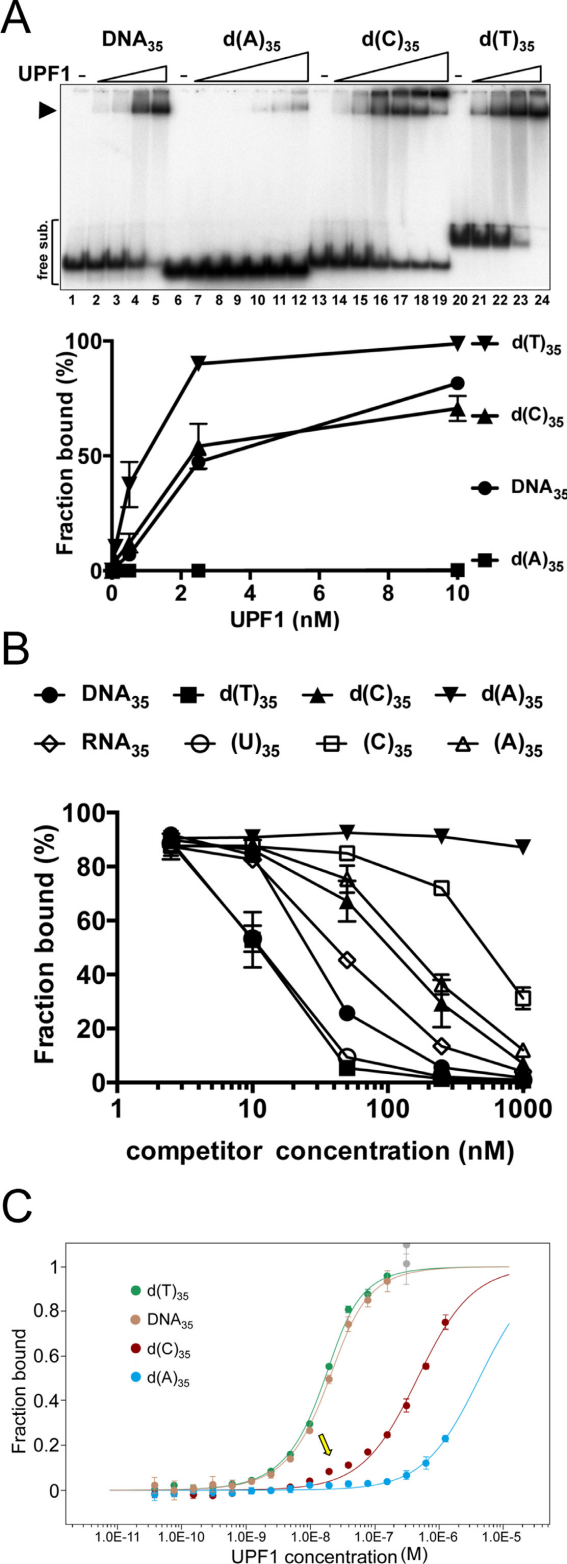
hUPF1 binding to 35 base DNA substrates. (**A**) EMSA analysis (0.1 nM substrate, 0.1–10 nM hUPF1 (DNA_35_ and d(T)_35_) and 0.1–100 nM hUPF1 d(A)_35_ and d(C)_35_,*n* = 3 experimental repeats, mean and standard deviation). Only the data for 0.1–10 nM UPF1 are plotted on the graph and the fraction bound was calculated from the sum of all shifted species. Data for RNA binding are shown in Supplementary Figure S7. (**B**) Graphed data from oligonucleotide competition assays (*n* = 4, mean and standard deviation). Reactions were assembled with ^32^P end-labeled substrate DNA_35_ (0.25 nM) and unlabeled (U)_35_, d(T)_35_, (A)_35_, d(A)_35_, (C)_35_, d(C)_35_, RNA_35_ or homologous DNA_35_ competitor (0, 2.5, 10, 50, 250 and 1000 nM) before addition of hUPF1 (10 nM) and EMSA. IC_50_ values of 11.5 nM, d(T)_35_; 12.3 nM, (U)_35_; 31.5 nM, DNA_35_; 50.4 nM, RNA_35_; 135.2 nM, d(C)_35_; 172 nM, (A)_35_; 952 nM, (C)_35_ and *d*(A)_35_, >> 1000 nM were determined by fitting to an IC50 equation. (**C**) hUPF1 binding to Alexa 647-labeled d(A)_35_, d(C)_35_, d(T)_35_ and DNA_35_ analyzed by MST using three independent dilution series of hUPF1. As in Figure 5, data for d(T)_35_ and DNA_35_ were analysed up to 156 nM hUPF1. Apparent *Kd* values of 6.01 ± 0.85 × 10^−9^ M, 9.8 ± 1 × 10^−9^ M, 4.52 ± 0.71 × 10^−8^ M and 4.2 ± 3.73 × 10^−6^ M for d(T)_35_, DNA_35_, d(C)_35_ and d(A)_35_ respectively were obtained. Supporting data is provided in Supplementary Figure S9.

To investigate hUPF1 nucleic acid interactions further we first tested binding to radiolabelled poly d(T) substrates in the absence of nucleotide cofactors using an electrophoretic mobility shift assay (EMSA, Figure 5A). hUPF1 bound oligo d(T)_15_ with apparent low affinity (lanes 1–4) relative to the other substrates tested. The apparent binding affinity increased significantly as the substrate length was increased from 15 to 45 d(T) residues (lanes 5–16) whereupon further increases in affinity were less pronounced (from 45 to 55 d(T) residues, as shown in the graph of the quantified data). For d(T)_*n*_ oligonucleotide up to 35 residues the major species observed was a single discrete protein-DNA complex, as indicated. For oligonucleotide d(T)_35_ at protein concentrations where most of the ssDNA substrate was bound, a minor fraction of the substrate (∼1%, lane 12) was retained in the well or appeared to migrate as a second species very close to the origin of the well. Further increases in protein concentration (≥10 nM) resulted in a progressive retention of the substrate at or close to the origin of the gel well (see Figure 6 below, for example). For oligonucleotides 45 residues in length or greater, retention of the substrate at or close to the origin of the gel was more pronounced at protein concentrations where most or all of the substrate was bound (lanes 15 and 16, 19 and 20). These data indicate that single hUPF1 binding events predominate on oligo d(T) substrate up to 35 residues in length, while higher protein concentrations promote protein multimerization on the nucleic acid substrate. The gel-shift data allow estimation of a *K*_d_ of ∼1 × 10^−9^ M for the hUPF1-d(T)_35_ interaction.

hUPF1 binding to substrates d(T)_35_, d(T)_25_ and d(T)_15_ 5΄ end-labeled with the fluorophore Alexa 647 was also characterised by microscale thermophoresis (MST). Binding to Alexa-d(T)_15_ displayed a sigmoidal dose response curve when plotted against log protein concentration, approaching saturation binding at 1250 nM hUPF1. From the data (Figure 5B and Supplementary Figure S5) a *K*_d_ of 3.94 ± 0.36 × 10^−8^ M for the hUPF1-d(T)_15_ interaction was obtained using the MO.Affinity Analysis software. Binding to the substrates Alexa-d(T)_25_ and Alexa-d(T)_35_ was observed at lower protein concentrations and displayed a biphasic transition at 156–313 nM hUPF1. This transition was interpreted as protein multimerization, which was also observed directly in the gel-shift experiments only after a single complex had formed on the majority of the substrate (Figure 5A). In order to determine binding constants, values affected by protein multimerization were discarded which permitted analysis with the automated algorithm in the MO.Affinity Analysis software to give apparent *K*_d_s of 4.05 ± 0.58 × 10^−9^ M and 5.98 ± 0.51 × 10^−9^ M for binding Alexa-d(T)_35_ and Alexa-d(T)_25_, respectively. Overall therefor, there is a high degree of agreement between observations of hUPF1-poly d(T)_*n*_ interactions observed in gel-shift and MST assays.

### Influence of nucleotide sequence on hUPF1 nucleic acid binding

Using oligonucleotide of 35 base unit length we compared hUPF1 binding to RNA and DNA homopolymers and RNA and DNA heteropolymers containing all four respective nucleobases (substrates DNA_35_ and RNA_35_) by EMSA. The mixed nucleobase sequence was the same for the corresponding DNA_35_ and RNA_35_ substrates and derived from the ssDNA sequence used in the helicase substrates characterized in Figure 2B. First, we addressed whether binding to RNA or DNA is altered substantially by the presence or absence of nucleotide cofactors (Supplementary Figure S6). Overall, the differences in binding extents observed in the presence of magnesium ions and non-hydrolsable nucleotides compared to their absence were small (∼2–8 fold), but binding in the presence of ATP/Mg^2+^ was substantially reduced. The relative magnitude of these differential effects is consistent with previous reports for binding of a (U)_15_ oligonucleotide to a truncated hUPF1 species (19). The same trend was observed regardless of the nucleic acid substrate tested (d(T)_35_, (U)_35_ and DNA_35_).

The data in Figure 6A show the results of EMSA experiments for hUPF1 binding to DNA substrates, while the results for binding to RNA substrates are shown in Supplementary Figure S7. On account of the lower apparent affinity observed for substrates with cytosine or adenine bases an extended titration series was used for these substrates and all shifted species were included in the calculation of fraction bound. Overall, the results did not reveal a clear preference for binding RNA compared to DNA or pyrimidine compared to purine polynucleotides. Considering the protein concentration required to bind 50% of the substrate (graphed data in Figure 6A and Supplementary Figure S7), the apparent affinity of hUPF1 for DNA_35_ was approximately 3 fold higher than for RNA_35_, and the affinity for d(T)_35_ ∼2-fold higher than for (U)_35_, both of which bound with higher affinity than DNA_35_ or RNA_35_. hUPF1 bound (A)_35_ with substantially higher affinity than (C)_35_ (∼5-fold), while binding to d(C)_35_ was substantially higher than binding to (A)_35_, (C)_35_ and d(A)_35_, where, for the latter, binding was barely detectable (Figure 6A, lanes 6–12). Overall, the order of apparent binding affinity observed by EMSA can be summarized as follows from highest to lowest: d(T)_35_>(U)_35_>d(C)_35_≈DNA_35_>RNA_35_>(A)_35_>(C)_35_>>d(A)_35_.

To verify the hUPF1-nucleic acid binding data in Figure 6A we performed competition binding experiments with radiolabelled DNA_35_ and increasing concentrations of unlabeled competitor nucleic acids. After resolution of the products by EMSA (Supplementary Figure S8) complex formation was quantified as shown in Figure 6B. Consistent with the data described above, d(T)_35_, and (U)_35_ were more effective competitors of hUPF1-DNA_35_ binding than the homologous competitor DNA (DNA_35_). All other competitors could be ranked in order of decreasing effectiveness as follows: RNA_35_>d(C)_35_>(A)_35_>(C)_35_>>d(A)_35_, where little competition was observed at the highest concentrations of d(A)_35_ (1000 nM, 100 fold molar excess over protein). Except for competitor d(C)_35_, there is good agreement with the binding data in Figure 6A. We note however that the hUPF1 binding to radiolabelled d(C)_35_ observed in Figure 6A, lanes 14–19, repeatedly displayed certain anomalous characteristics compared to the other substrates tested: As the hUPF1 protein concentration was increased there was a greater tendency for retention in the gel well and there was little or no increased substrate binding observed when the protein concentration was increased from 10 to 100 nM (lanes 17–19).

Given the large differences in apparent binding affinity for the nucleic acid substrates observed by EMSA, and in particular the DNA polymers, we assayed binding to Alexa 647-labeled d(A)_35_, d(C)_35_, d(T)_35_, and DNA_35_ in parallel in one experimental group by MST. d(T)_35_ and DNA_35_ exhibited a biphasic binding curve with a transition above ∼156 nM (Figure 6C and Supplementary Figure S9). As above, apparent *K*_d_ values of 6.01 ± 0.85 × 10^−9^ M and 9.8 ± 1 × 10^−9^ M for d(T)_35_ and DNA_35_ respectively were determined using the automated algorithm in the MO.Affinity Analysis software, after discarding the values obtained with hUPF1 concentration >156 nM that were affected by protein multimerisation.

Analysis of the MST observed with the Alexa 647-labeled d(A)_35_ and d(C)_35_ substrates indicated low affinity interactions with hUPF1 with apparent *K*_d_ values of 4.2 ± 3.73 × 10^−6^ and 4.52 ± 0.71 × 10^−8^ derived using the the MO.Affinity Analysis software (Figure 6C). However, close inspection of the binding curves also indicate a biphasic transition (indicated with the arrow in Figure 6C) with the first transition displaying a low amplitude (Supplementary Figure S9), much lower in the case of Alexa 647 labeled d(A)_35_. The first low amplitude phase suggests a strong affinity toward the d(A)_35_ and d(C)_35_ substrates, similar in magnitude to that observed for substrates d(T)_35_, and DNA_35_, but the binding data do not allow extraction of a reliable *K*_d_ value in the low amplitude phase. Taken together with the observations in Figure 6A and B, the data indicate that there could be isoforms of the d(A)_35_ and d(C)_35_ substrates and that hUPF1 reacts with high affinity to one and not another. In the case of the d(A)_35_ substrate the high affinity species is rare. This hypothesis is consistent with the observations in the EMSA, Figure 6A, lanes 13–19, as noted above, where a significant fraction of the d(C)_35_ substrates fails to bind hUPF1 as its concentration is increased.

Overall, there is a consistency between protein-nucleic acid interactions probed by EMSA, in competition binding experiments and also MST in the case of the DNA substrates. Furthermore hUPF1–ssNA binding affinity correlates absolutely with the ability of hUPF1 to unwind helicase substrates (Figure 2 and Supplementary Figure S10), indicating that ATP/Mg^2+^ does not affect such interactions.

### hUPF1 shows little affinity or specificity for ds-, G4 and triplex DNA secondary structures

**Figure 7.**
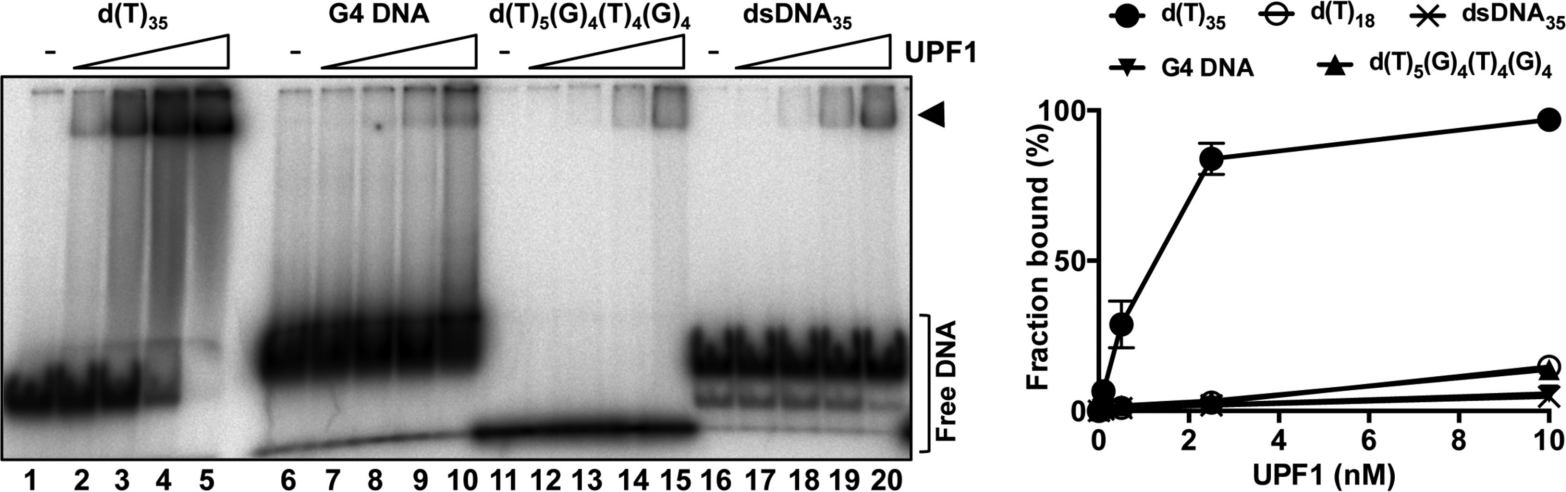
hUPF1 binds with low affinity to dsDNA and G4 DNA. hUPF1 binding (0.1 nM substrate, 0.1-10 nM UPF1) to ^32^P end-labeled substrate d(T)_35_ was compared in parallel with G4 DNA, the 17 base single-stranded precursor of the G4 DNA substrate (5΄-dTTTTTGGGGTTTTTGGGG), a 35 base pair dsDNA substrate consisting of the ssDNA substrate analyzed above (DNA_35_) annealed to its complementary strand and a d(T)_18_ oligonucleotide (not shown in the gel image but data included in the graph on the right; *n* = 3 experimental repeats, mean and standard deviation). The DNAs with inter-base hydrogen bond mediated secondary structure (G4 and dsDNA) bound with lower affinity than all ssDNA substrates tested. hUPF1 also failed to bind triplex DNA (Supplementary Figure S11).

The results described above demonstrate that full-length hUPF1 binds avidly to single-stranded nucleic acids in a length-dependent manner that is significantly influenced by nucleobase composition. Furthermore, the enzyme is capable of resolving a variety of B-form (ssDNA forks and partially single- and double-stranded substrates) as well as non-B structures (triplex and G-quadruplex). We next tested whether hUPF1 could bind to dsDNA and non-B form DNA structures stabilized by inter-base hydrogen bonding. As shown in Figure 7, minimal complex formation was observed between hUPF1 and a G4 substrate with two sets of four G4 tetrads (lanes 6–10). We have previously shown that the helicase hPIF1 binds avidly to this structure (30). The extent of G4 DNA binding observed was similar to binding to the 35 base pair dsDNA substrate (lanes 16–20), but less than that observed with its single-stranded precursor (5΄-dTTTTTGGGGTTTTGGGG, lanes 11–15), which bound with equivalent affinity to the substrate d(T)_18_ analysed in parallel. Similarly, hUPF1 failed to bind the triplex substrate TripT0 (used in Figure 3) with a 21 base triplex motif (Supplementary Figure S11).

## DISCUSSION

Previous studies on hUPF1-nucleic acid interactions have focused largely on its ability to translocate on RNA and unwind RNA:DNA hybrids and have exploited truncated species encompassing the helicase core, which itself demonstrates a high degree of processivity in ssRNA and ssDNA translocation (27,34). Here we have analysed full-length hUPF1 nucleic acid binding and unwinding in detail for the first time. We show that hUPF1 interacts primarily with single-stranded nucleic acids with no clear preference for binding RNA compared to DNA or purine compared to pyrimidine polynucleotides. The data however demonstrate a highly pronounced nucleobase bias in hUPF1-NA interactions, which spans several orders of magnitude of apparent affinity. The enzyme is also capable of resolving non-B DNA configurations including triplex and G quadruplex (G4) DNA. Without detectable secondary structure specific NA binding it is likely that these substrates require a 5΄-ssNA component for targeting and initiation of unwinding.

The interaction of DNA and RNA helicases with ssNA polymers is widely regarded as being uninfluenced by the identity of the nucleobases, that is it is sequence independent (35). This is supported by all available high-resolution structural data. SF1 and SF2 helicases make extensive substrate phosphodiester backbone contacts while non-specific stacking and hydrophobic interactions with the bases are more common in SF1 helicases. Base sequence effects, not otherwise seen in ensemble experiments, are frequently observed in single molecule unwinding assays as periodic stepping and pausing behavior for several helicases (36–38) including hUPF1 (34). However, in all cases the behavior has been attributed to dsDNA secondary structure and its thermodynamic stability, while direct sensing of the nucleobases (sequence specific interactions) has been discounted. There are notable examples nonetheless of helicases that are sequence-specific NA binding proteins or whose activity is altered when they encounter a specific nucleotide sequence (e.g. the SF3 viral helicases E1 and T-antigen, bacterial RecBCD and RNA helicase A (RHA), (39–41). Importantly though, in each of these cases a separate subunit or a distinct functional module, which is not a direct extension of the helicase core that translocates on ssNA, is responsible for sequence specific NA recognition.

Our data show that the nucleobases can have a profound influence on the affinity of hUPF1 for ssNA polymers with no clear bias for purines compared to pyrimidines, although binding to d(T)_35_ (or (U)_35_) compared to d(A)_35_ polymers is at least two orders of magnitude higher in affinity. It is unclear how hUPF is sensitive to base sequence and whether the helicase core or auxiliary domains are responsible. However, it is notable that while yeast and human UPFHD-RNA-ADP:AlF_4_^−^ structures show extensive phosphodiester backbone but minimal base contacts, nucleobase interactions are significant in the extension to the RNA binding channel observed in the yeast Upf1-RNA-ADP:AlF_4_^−^ structure that includes the N-terminal CH domain (19). Given the high sequence similarity between UPF1 orthologs it is likely that the hUPF1 ssNA binding channel also extends beyond the helicase core and could mediate a more extensive wrapping of ssNAs engaged in nucleobase specific interactions on the protein surface. Our oligo d(T) binding data (Figure 5) are at least consistent with an extended ssNA binding channel and demonstrate the formation of a single discrete hUPF1-NA species with increasing affinity on oligonucleotides of increasing length. Similar observations have been made with yeast Upf1, which forms a single RNA-protein complex with oligonucleotides up to 34 nucleotides long. Furthermore, N-terminal domain mutants that alter RNA-protein complex formation *in vitro* affect nonsense-mediated decay *in vivo* (9), indicating the functional importance of such interactions.

Helical ssNA forms stabilized by base stacking interactions have been observed in crystal structures but evidence for their existence in solution is based on indirect observations and is less conclusive. However, it has been proposed that poly d(T) and poly (U) exhibit negligible base stacking while poly-d(C), -(C), -d(A) and -(A) all display evidence of a transition to a helical structure in solution. Furthermore, the poly d(A) helix is considered to be more stable than the poly d(C) helix (42–44). Although nucleobase sensing by helicases during translocation is not without precedent (45), our hUPF1 ssNA binding analysis cannot differentiate between direct sensing of the nucleobase identity or sensitivity to ssNA secondary structure. Nonetheless, our results raise the intriguing possibility that the preferred NA substrate for hUPF1 binding is an extended (∼35 residues) ssNA chain with minimal secondary structure induced by base stacking or hydrogen bonding. This requirement may not necessarily be a universal property of helicases since dengue virus NS3 helicase binds AGUUG repeats with ten times higher affinity than poly (A) and 100 times higher affinity than poly (U) (46). Also, our unpublished observation show that the hPIF1 helicase binds d(C)_35_ with higher affinity than d(T)_35_. hUPF1 sequence dependent NA interactions, whether due to direct nucleobase interactions or sensing or secondary structure, could have biological relevance since homopolymeric nucelotides tracts are common in the human genome and transcriptome.

The current favoured model for NMD regulation by hUPF1 is that mRNA binding is regulated by ATP hydrolysis, which serves to dissociate non-productive mRNA binding (47). Yeast Upf1 (48) and the hUPFHD (13) both show reduced RNA binding affinity in the presence of ATP and our observations with full-length hUPF1 are consistent with this. Using mononuclotide polymers we revealed a sequence bias in hUPF1 NA interaction showing a preference for binding d(T)_35_ or (U)_35_ compared to DNA and RNA heteroploymers (Figure 6). mRNA 3΄ untranslated regions (3΄UTRs) are A/U rich elements (AREs) composed of AUUUA repeats and polyU tracts (49) and many RNA binding proteins important for the regulation of RNA stability interact with AREs. *In vivo* binding data show that hUPF1 associates with 3΄UTRs (50–54) and this association is an initiating event in NMD and a reliable indicator of mRNAs destined for NMD (47). A key factor that determines this distribution is believed to be elongating ribosomes that displace hUPF1 from 5΄UTRs and coding sequences (51). However, our data indicate that their U-rich nature is important for hUPF1 recruitment and are supported by *in vivo* experiments showing that hUPF1 preferentially cross-links to U nucleotides (51). *In vivo*, UPF1 phosphorylation also occurs when it is bound to 3΄UTRs (47) and it is possible that this posttranslational activity could modulate hUPF1-ssNA interactions further.

The role of hUPF1 in genome stability is poorly understood although observations support a direct role at the elongating replication fork. We have shown that hUPF1 can resolve two forms of non-B-form DNA secondary structure, G4 and triplex DNA, both of which can stall replication forks and are implicated in genome instability (17,55,56). How the eukaryotic replicative helicase complex (GINS-MCM-Cdt45 or CMG complex, ref. 57) responds when non-B form DNA is encountered is unknown. However, the model viral hexameric replicative helicase SV40 T-antigen unwinds G4 (58) but not triplex (59) DNA substrates *in vitro*. A number of helicases required for genome stability resolve triplex DNA structures in an ATP-dependent manner including WRN, BLM (60) and RHA (32) that move in the 3΄–5΄ direction and FANCJ (61) and ChlR1 (62) that move 5΄–3΄. WRN, BLM and FANCJ will also resolve G4 DNA structures *in vitro* (63). Bi-polar helicases are rare. The observation that hUPF1 can resolve triplex DNA with either a 5΄ or 3΄ ssDNA extension, but only dsDNA or G4 DNA with a 5΄ tail, in an ATP-dependent manner is novel and may indicate an important role in their resolution.

Although generally regarded as sequence independent, substrate recognition by helicases can be structure dependent. Several helicases including those of the RecQ family are enriched at potential G4 forming sites in intact cells but so far only hPIF1 (30), WRN and BLM (64) have showed specificity for G4 DNA binding *in vitro*, while ChlR1 has been shown to bind triplex DNA (62). We were unable to detect hUPF1 binding to DNA secondary structures stabilized by inter-base hydrogen bonding, ds-, G4 and triplex DNA. The data indicate that, as in mRNA recognition, ssDNA length and sequence are the primary factors influencing hUPF1 substrate choice. Although the nature and functional consequence of the physical coupling between hUPF1 and pol δ are unknown it is likely to stabilize hUPF1 binding to ssDNA. Helicase-and polymerase motors are often coupled at the replication fork (65), serving to mutually increase their forward velocity while the helicase provides the potential to resolve obstacles such as non-B DNA secondary structure and bound proteins (66). In the future it will be important to understand the nature of these couplings and what determines the context in which the myriad of helicases with apparently overlapping functions act. Our data indicate that nucleotide sequence-dependent effects should be considered further.

## Supplementary Material

Supplementary Data
