# Supplementary material for: DNA substrate recognition and processing by the full-length human UPF1 helicase
Source: Nucleic Acids Res. 2017 May 24;45(12):7354–66. doi: 10.1093/nar/gkx478 (PMC5499549; doi:10.1093/nar/gkx478)
Supplement: Supplementary Data [file gkx478_Supp.pdf]

Figure S1. Nucleic acid substrates used in this study.

Partially single- and double-stranded helicase substrates:

5′d(T)$_{55}$ds20

```
5′TTTTTTTTTTTTTTTTTTTTTTTTTTTTTTTTTTTTTTTTTTTTTTTTTTTTTTCGAATTCGAGCTCGGTACCC
                                                       GCTTAAGCTCGAGCCATGGG-5′
```

DNAd(A)$_{50}$

```
5′GGGAAAAAAAAAAAAAAAAAAAAAAAAAAAAAAAAAAAAAAAAAAAAAAAAACGAATTCGAGCTCGGTACCC
                                                     GCTTAAGCTCGAGCCATGGG-5′
```

DNAd(A)$_{33}$

```
5′GGGAAAAAAAAAAAAAAAAAAAAAAAAAAAAAAAAACGAATTCGAGCTCGGTACCC
                                    GCTTAAGCTCGAGCCATGGG-5′
```

DNA:DNA

```
5′-GGGCTCATCGACCCTATCAGCACAAGCAACCAATCGGTTCGACACTCATACTGGCCGAATTCGAGCTCGGTACCC
                                                          GCTTAAGCTCGAGCCATGGG-5′
```

RNAd(A)$_{50}$

```
5′GGGAAAAAAAAAAAAAAAAAAAAAAAAAAAAAAAAAAAAAAAAAAAAAAAAACGAAUUCGAGCUCGGUACCC
                                                     GCTTAAGCTCGAGCCATGGG-5′
```

RNAd(A)$_{33}$

```
5′GGGAAAAAAAAAAAAAAAAAAAAAAAAAAAAAAAAACGAAUUCGAGCUCGGUACCC
                                    GCTTAAGCTCGAGCCATGGG-5′
```

RNA:DNA

```
5′-GGGCUCAUCGACCCUAUCAGCACAAGCAACCAAUCGGUUCGACACUCAUACUGGCCGAAUUCGAGCUCGGUACCC
                                                          GCTTAAGCTCGAGCCATGGG-5′
```

Tetramolecular G quadruplex DNA forming oligonucleotides:

5′T55G4

5′-TTTTTTTTTTTTTTTTTTTTTTTTTTTTTTTTTTTTTTTTTTTTTTTTTTTTTTT**GGGG**TT

5′T25G4

5′-TTTTTTTTTTTTTTTTTTTTTTTTT**GGGG**TT

5′T10G4

5′-TTTTTTTTTT**GGGG**TT

G4T55-3′

5′-TT**GGGG**TTTTTTTTTTTTTTTTTTTTTTTTTTTTTTTTTTTTTTTTTTTTTTTTTTTTTT

5′T55G4X2

5′-TTTTTTTTTTTTTTTTTTTTTTTTTTTTTTTTTTTTTTTTTTTTTTTTTTTTTTT**GGGG**TTTT**GGGG**

5′T5G4X2

5′-TTTTT**GGGG**TTTT**GGGG**

Duplex DNA forming oligonucleotides (substrate dsDNA$_{35}$):

```
5′-CACAAGCAACCAATCGGTTCGACACTCATACTGGC
   GTGTTCGTTGGTTAGCCAAGCTGTGAGTATGACCG-5′
```

Triplex forming oligonucleotides:

Duplex

5′–ACGTTCTAGAGCGCGCCACCCAGCAAACAACACT**GGGGGAGGGGGACGGTGAAG**GCCAAGTTCCCCGCCAGTCGGCTTAGATCTAGATGCA
TGCAAGATCACGCGCGGTGGGTCGTTTGTTGTGA**CCCCCTCCCCCTGCCACTTC**CGGTTCTTGGGGCGGTCAGCCGAATCTAGATCTACGT–5′

Trip3′T55

5′–**GGAAGCGGTAGGGGGAGGGGG**TTTTTTTTTTTTTTTTTTTTTTTTTTTTTTTTTTTTTTTTTTTTTTTTTTTTTTT

Trip5′T55

5′–TTTTTTTTTTTTTTTTTTTTTTTTTTTTTTTTTTTTTTTTTTTTTTTTTTTTTTT**GGAAGCGGTAGGGGGAGGGGG**

TripT0

GGAAGCGGTAGGGGGAGGGGG

Supplementary Figure S1. Nucleic acid substrates used in this study. Partially single- and double-strand substrate sequences are given. The RNA polynucleotides were generated by run-off transcription and the three 5′G residues are derived from the T7 promoter sequence used. The substrate ds20d(T)$_{55}$3′ had a 3′ d(T)$_{55}$ extension to the top strand and annealed to the same 20 bp oligonucleotide as all other substrates shown. The sequences of the single-stranded precursor oligonucleotides used to generate the tetramolecular G4 DNA substrates are given. The duplex sequence generated by PCR for formation of the triplex DNA substrates is shown along with the third strands for triplex formation. The triplex forming sequences are in bold. See materials and methods for further details on generation of each substrate.

Figure S2. Optimisation of hUPF1 helicase activity.

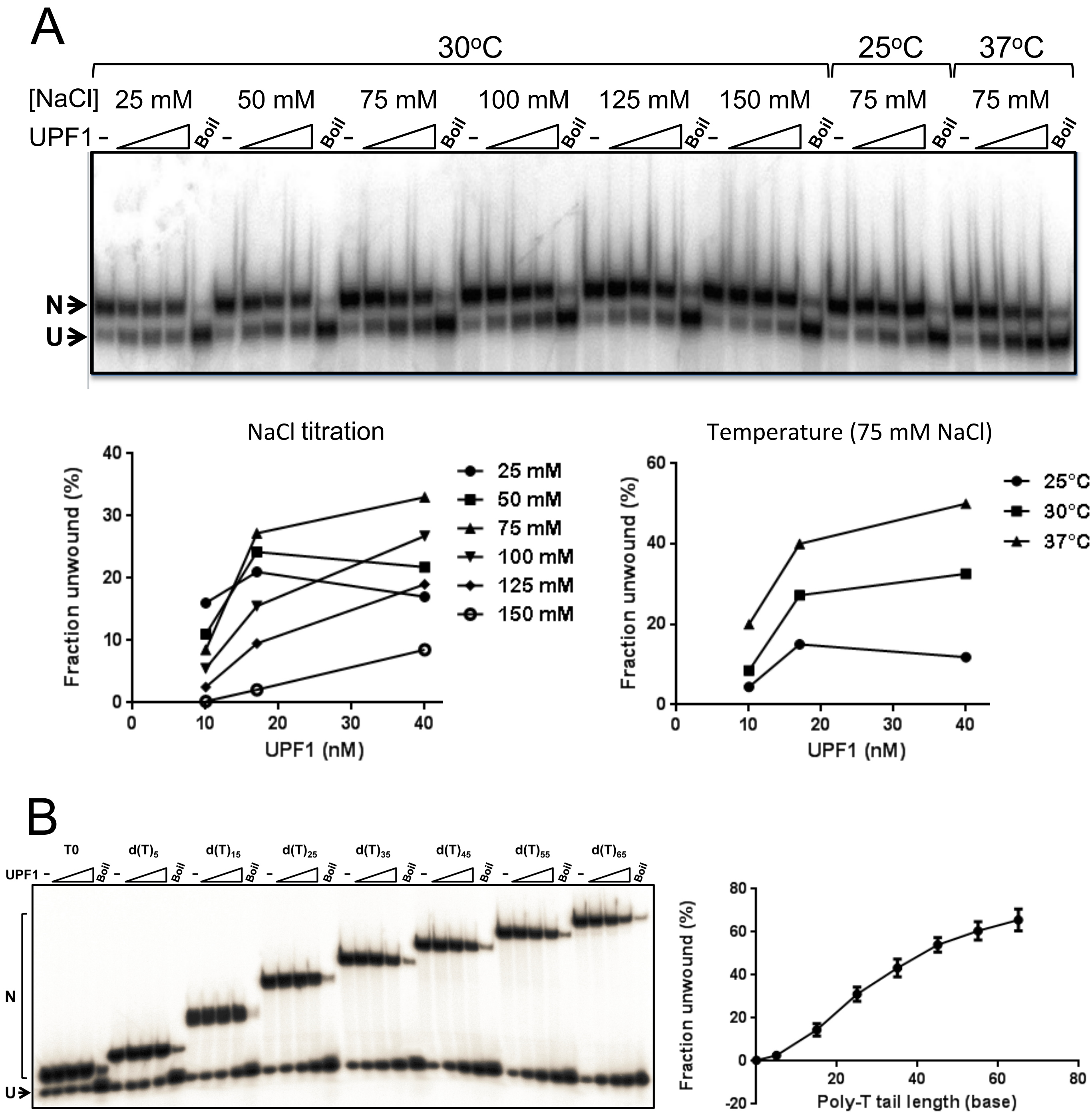

Supplementary Figure S2. Optimisation of hUPF1 helicase activity. (**A)** Initial experiments determined a pH optimum of ~7.2 in 25 mM HEPES-NaOH buffer (not shown). Further assays (10-40 nM hUPF1, 0.2 nM substrate 5′d(T)$_{55}$ds20, long strand labeled) indicated an optimum salt concentration of 75 mM for unwinding, with significant inhibition at concentrations greater than 100 mM. A temperature of 37ºC was optimal for unwinding at 75 mM NaCl (*n=2*). (**B**) Unwinding as a function of 5′-d(T) tail length (10-40 nM hUPF1, 0.2 nM substrate 20 base oligonucleotide labeled, *n=2*). All substrates employed the same 20 bp duplex region (Supplementary Figure S1) with 5′-d (T) ssDNA extensions from 5 to 65 nucleotides. Almost no unwinding was observed for the substrate with a 5 nucleotide 5′extension. After reaching 45 5′-d(T) residues further increases in unwinding efficiency became less pronounced as the 5′-d(T) tail was increased.

Figure S3. hUPF1 unwinding of a fork-like DNA substrate with ssDNA arms.

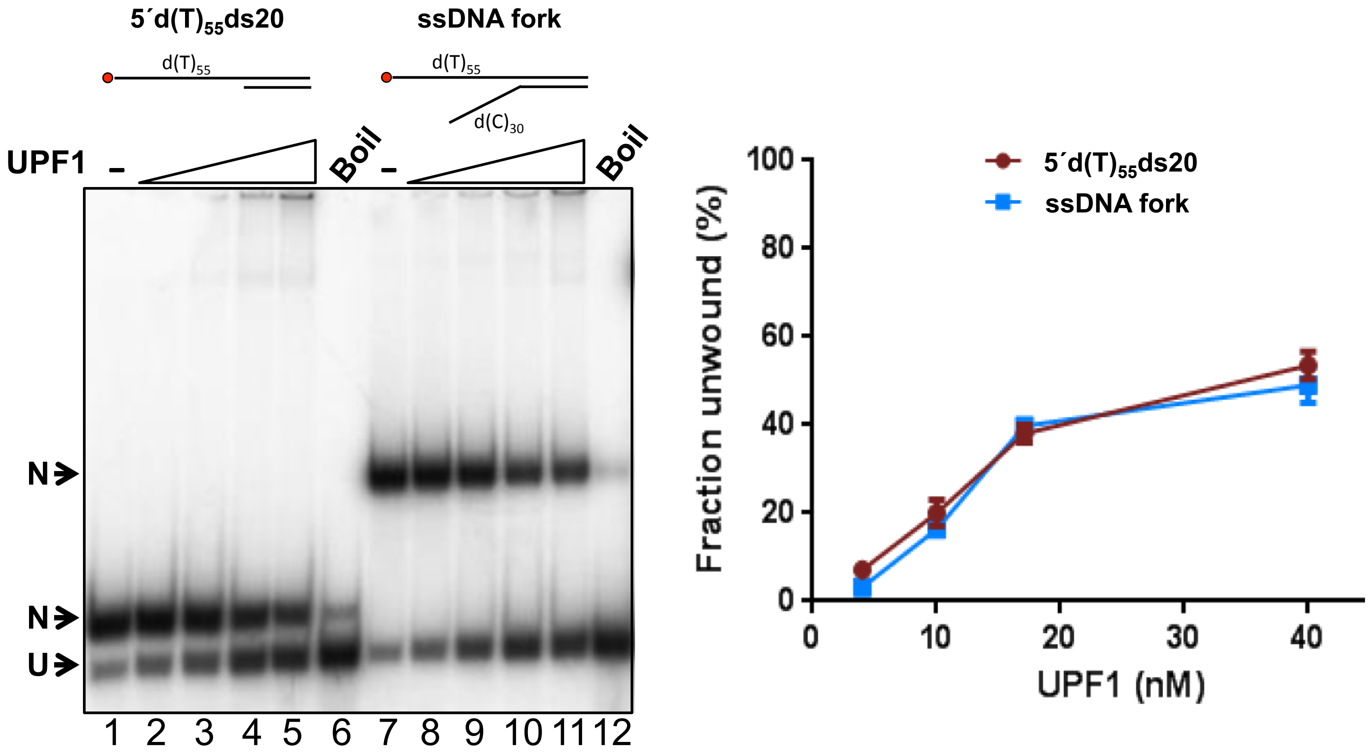

Supplementary Figure S3. hUPF1 unwinding of a fork-like DNA substrate with ssDNA arms. Unwinding reactions were performed under standard conditions (0.2 nM substrate, 4, 10, 17 and 40 nM hUPF1) with substrate 5´d(T)$_{55}$ds20 (lanes 1-6) and a ssDNA forked duplex with an additional 3´d(C)$_{30}$ extension to generate the fork (lanes 7-12). The graph to the right shows the quantified results from *n=4* repeats. No significant differences in unwinding extents were observed between the two substrates.

Figure S4. ATP is required for hUPF1 to unwind triplex DNA.

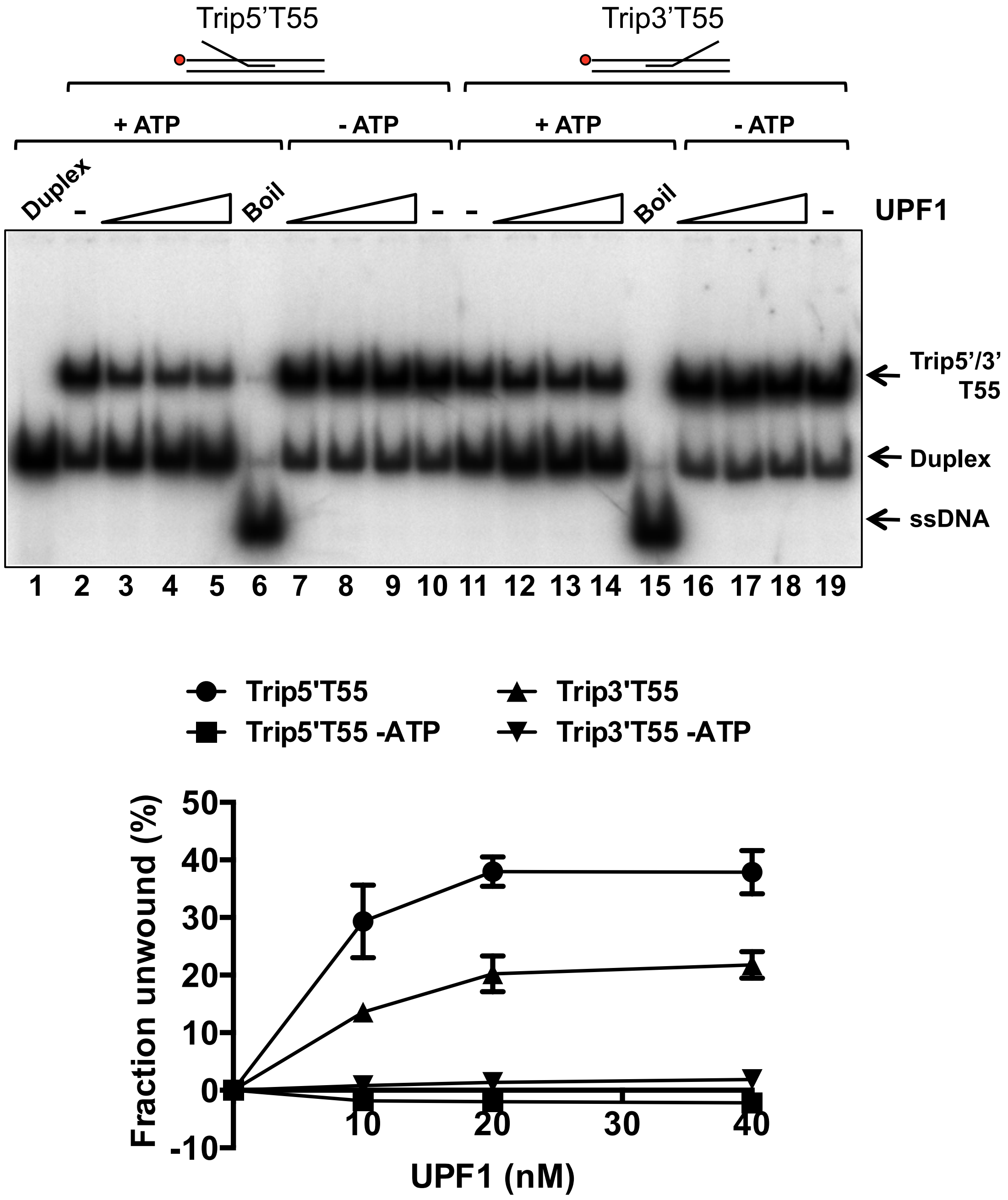

Supplementary Figure S4. ATP is required for hUPF1 to unwind triplex DNA. Reactions were performed as described in materials and methods (0.2 nM substrate, 10-40 nM hUPF1). As shown in Figure 3 (main manuscript), substrate Trip5´T55 was resolved effectively by hUPF1, as was Trip3´T55 but at ~60% efficiency compared to substrate Trip5´T55. In the absence of ATP there was minimal unwinding of substrate Trip5´T55. However, as noted previously for reactions containing Trip3´T55, the variant hUPF1 K498A (KA-UPF1) and ATP, in the absence of ATP hUPF1 appeared to stabilize substrate Trip5´T55 against non-enzymatic dissociation (~3% less non-enzymatic dissociation in the presence of hUPF1, see graphed data, *n=3*, mean and standard deviation).

Figure S5. MST analysis of the interaction between UPF and DNA substrates $d(T)_{35}$, $d(T)_{25}$ and $d(T)_{15}$

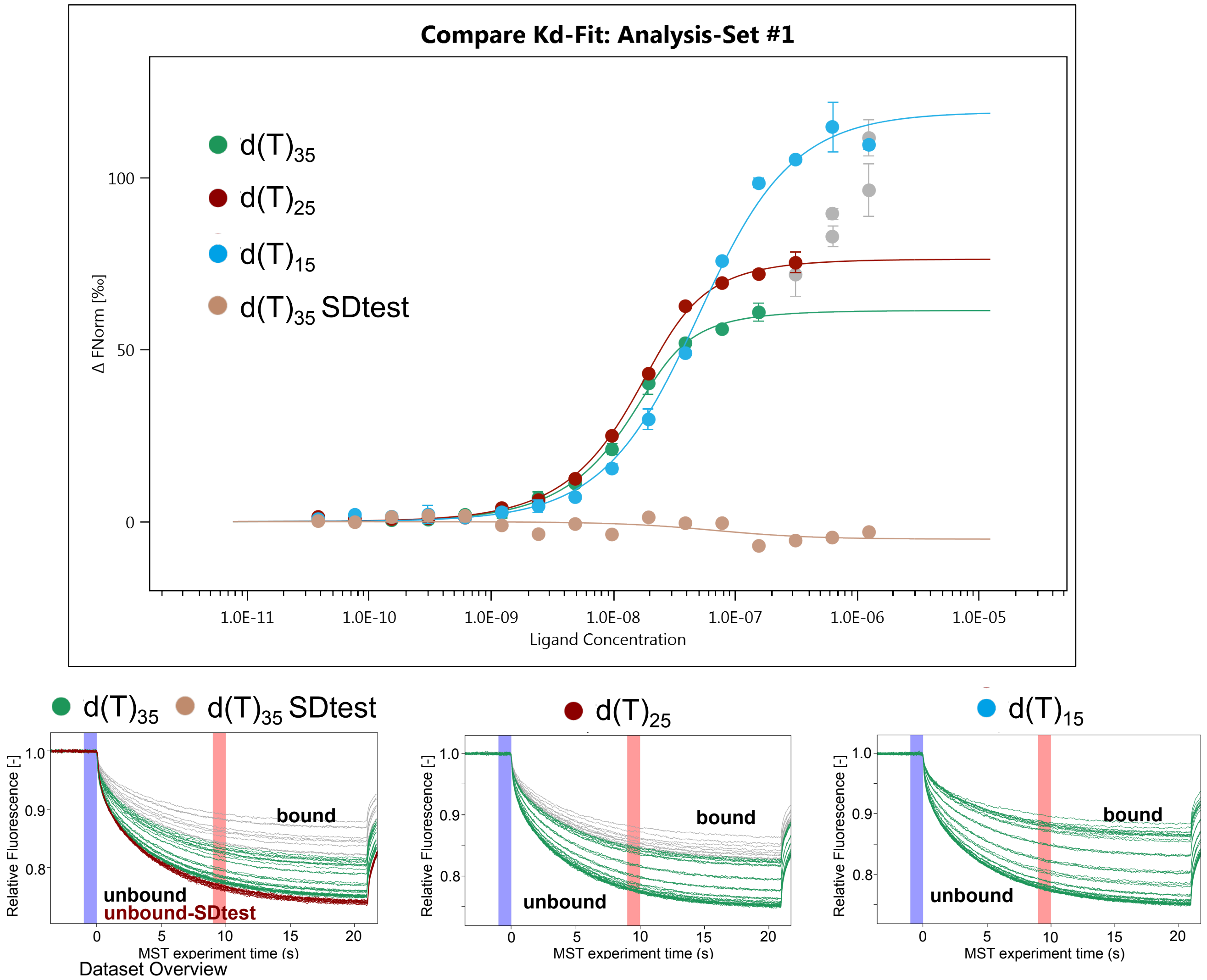

Dataset Overview

Name:	UPF Tray	UPF Tray	UPF Tray	UPF Tray
Graph Color:	•	•	•	•
Target Name:	T35	T25	T15	T35SDtest
Target Concentration:	20 nM	20 nM	20 nM	20 nM
Ligand Name:	UPF	UPF	UPF	UPF
Ligand Concentration:	156 nM to 0.0381 nM	313 nM to 0.0381 nM	1.25E+03 nM to 0.0381 nM	1.25E+03 nM to 0.0381 nM
n:	3	3	3	1
Comments:				
Excitation Power:	20%	20%	20%	20%
MST Power:	40%	40%	40%	40%
Temperature:	22.0°C	22.0°C	22.0°C	22.0°C
Kd:	4.0552E-09	5.9828E-09	3.9383E-08	5.8548E-08
Kd Confidence:	± 5.8436E-10	± 5.1056E-10	± 3.5562E-09	± 9.5645E-08
Response Amplitude:	61.328205	76.228086	118.9854	5.0738879
TargetConc:	2E-08[Fixed]	2E-08[Fixed]	2E-08[Fixed]	2E-08[Fixed]
Unbound:	775.6	775.85	774.88	769.09
Bound:	836.92	852.08	893.86	764.01
Std. Error of Regression:	1.0472669	1.0117413	2.7877098	2.1500001
Reduced χ^2:	2.3145373	11.358445	10.895597	
Signal to Noise:	64.149532	81.906969	45.847966	2.5349905

Supplementary Figure S5. hUPF1 interactions with Alexa 647 labeled $d(T)_{35}$, $d(T)_{25}$ and $d(T)_{15}$ analysed by MST. Reactions were performed as described in materials and methods, main manuscript (*n=3* measurements). A graph of Baseline Corrected Normalized Fluorescence [$^0/_{00}$] plotted against hUPF1 concentration (0.0381-1250 nM) is shown. Curve fitting was performed using the automated algorithm in the MO.Affinity Analysis software ($d(T)_{35}$, 0.0381-156 nM hUPF1; $d(T)_{25}$ 0.0381-313 nM hUPF1 and $d(T)_{15}$ 0.0381-1250 nM hUPF1). A representative data set for the $d(T)_{35}$ sample denaturation test (SDtest) is also shown. MST traces were analysed at an MST on time of 10 seconds. Grey data points and MST curves are for values discarded to allow curve fitting.

Figure S6. Modulation of hUPF1 ssNA binding by magnesium ions and nucleotide cofactors.

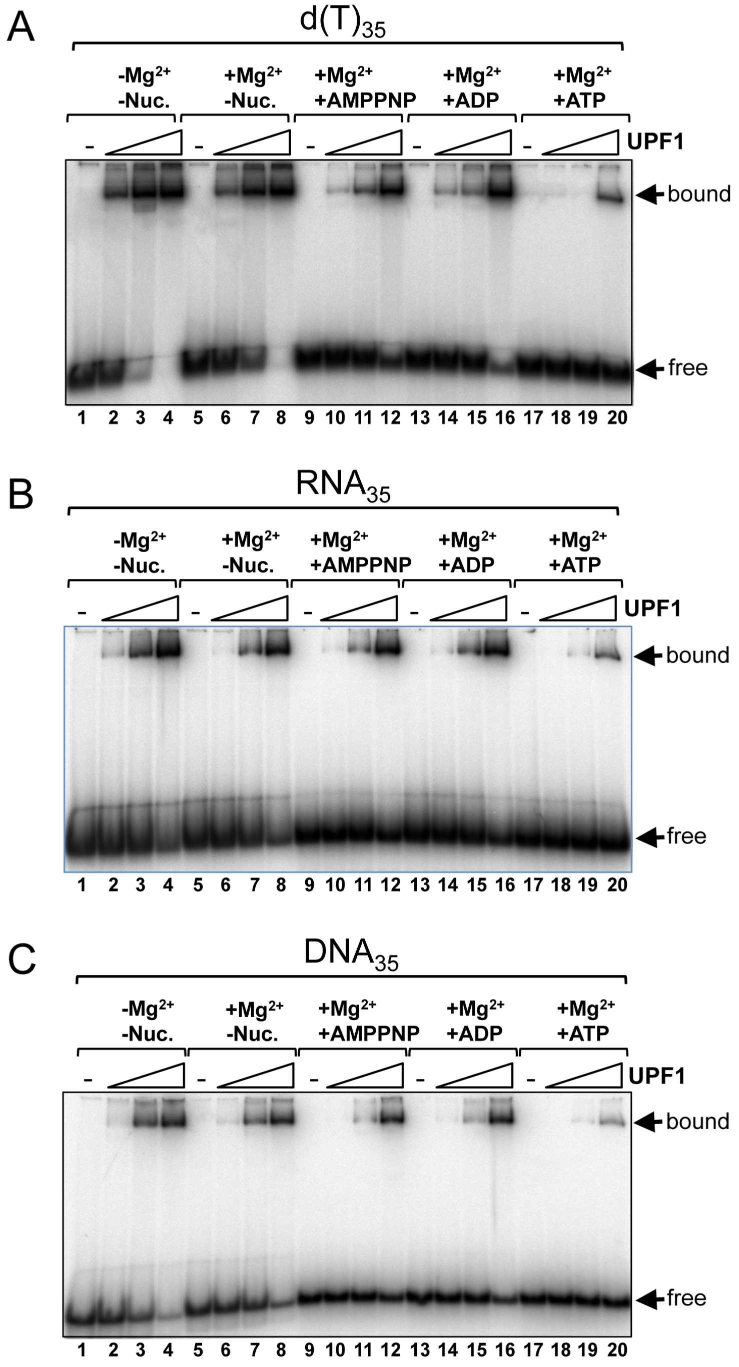

Supplementary Figure S6. Modulation of hUPF1 ssNA binding by magnesium ions and nucleotide cofactors. Reactions were assembled as described in materials and methods (0.1 nM substrate, 0.5, 2.5 and 10 nM hUPF1) for the three substrates $d(T)_{35}$, RNA_{35} and DNA_{35} and analysed by EMSA. Each cofactor ($MgCl_2$ and ATP, ADP and AMPPNP (adenylyl-imidodiphosphate) was present at 5 mM as indicated (-nuc. = no nucleotide cofactor). Cofactor and $MgCl_2$ ions altered affinity in the order no-cofactor/$MgCl_2$>$MgCl_2$>ADP/$MgCl_2$>AMPPNP>ATP/$MgCl_2$. Similar results were reported for hUPF1 helicase domain (residues 295-914, ref. 19, main manuscript).

Figure S7. hUPF1 binding to ^{32}P-end labeled 35 base RNA substrates.

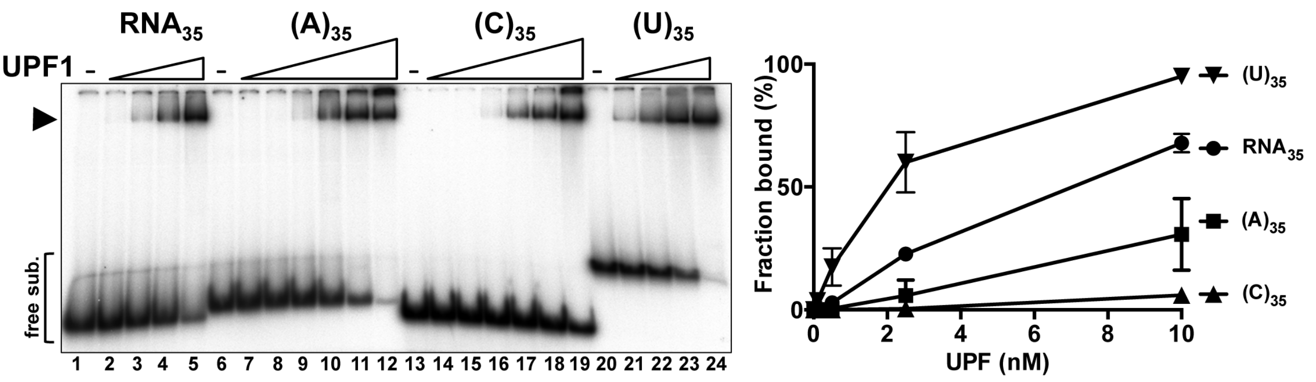

Supplementary Figure S7. hUPF1 binding to ^{32}P-end labeled 35 base RNA polymers. Reactions were performed in parallel with the corresponding DNA substrates shown in Fig. 6A, main manuscript. 0.1 nM substrate, 0.1, 0.5, 2.5 and 10 nM hUPF1 (RNA_{35} and $(U)_{35}$) and 0.1, 0.5, 2.5, 10. 40 and 100 nM UPF1 ($(A)_{35}$ and $(C)_{35}$). The graph to the right ranks the affinity in the order $(U)_{35}$>RNA_{35}>$(A)_{35}$>$(C)_{35}$; only the data for 0.1 to 10 nM UPF1 have been plotted. Error bars are the standard error of *n=3* measurements.

Figure S8. hUPF1 nucleic acid competition binding experiments.

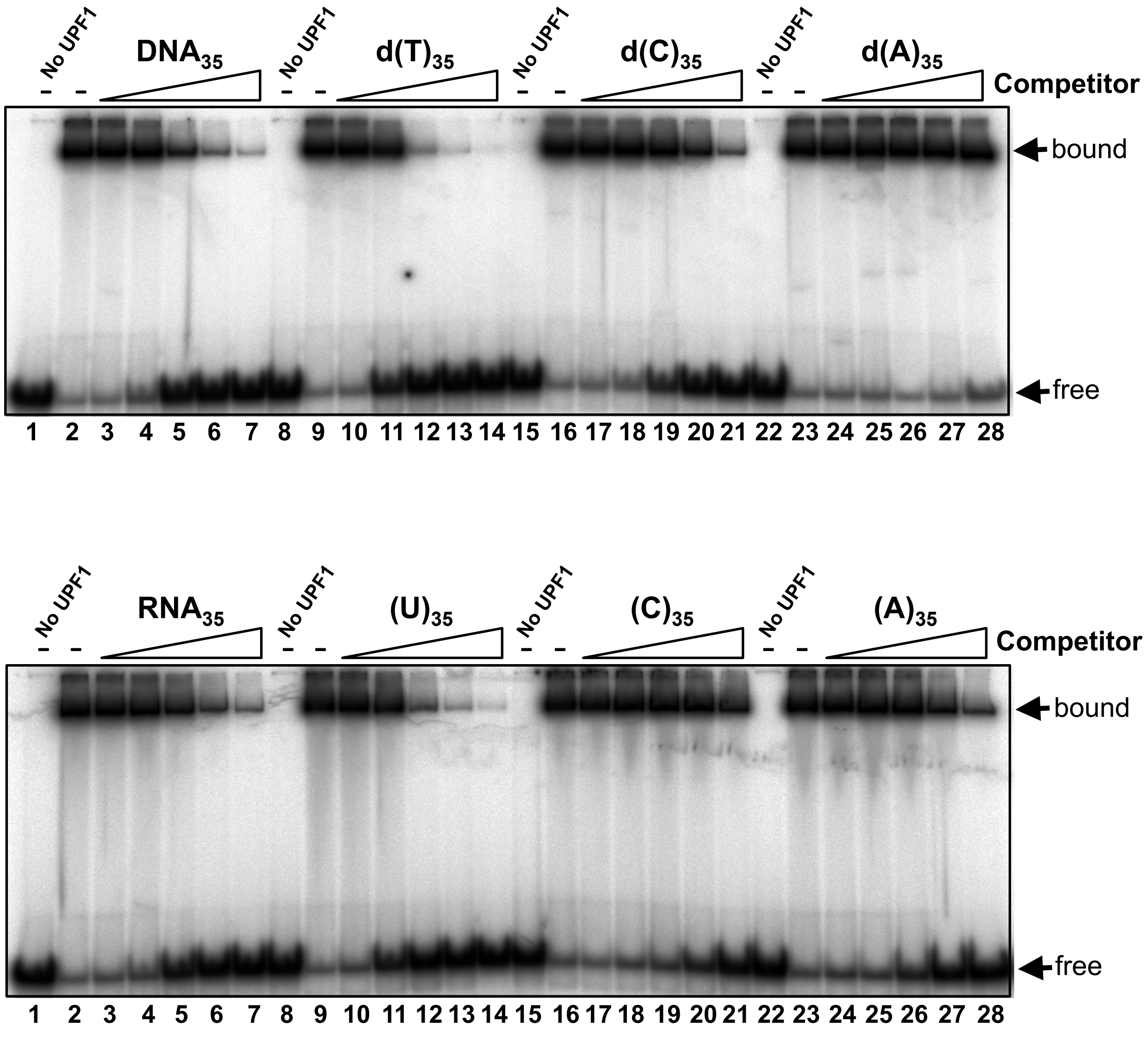

Supplementary Figure S8. hUPF1 nucleic acid competition binding experiments. Reactions were assembled under standard conditions (*n=4*) with 0.25 nM ^{32}P end-labeled DNA_{35} substrate, and the indicated unlabeled competitor RNA and DNA oligonucleotides (0, 2.5, 10, 50 250 and 1000 nM) before the addition of protein (10 nM hUPF1 final, except lanes 1, 8, 15, and 22, free DNA control). Products were resolved by EMSA and the fraction of labeled substrate bound determined by quantification of phosphorimages (Figure 6 main manuscript).

Figure S9. MST analysis of the interaction between hUPF1 and DNA substrates d(A)$_{35}$, d(C)$_{35,}$ d(T)$_{35}$ and the DNA$_{35}$ heteropolymer.

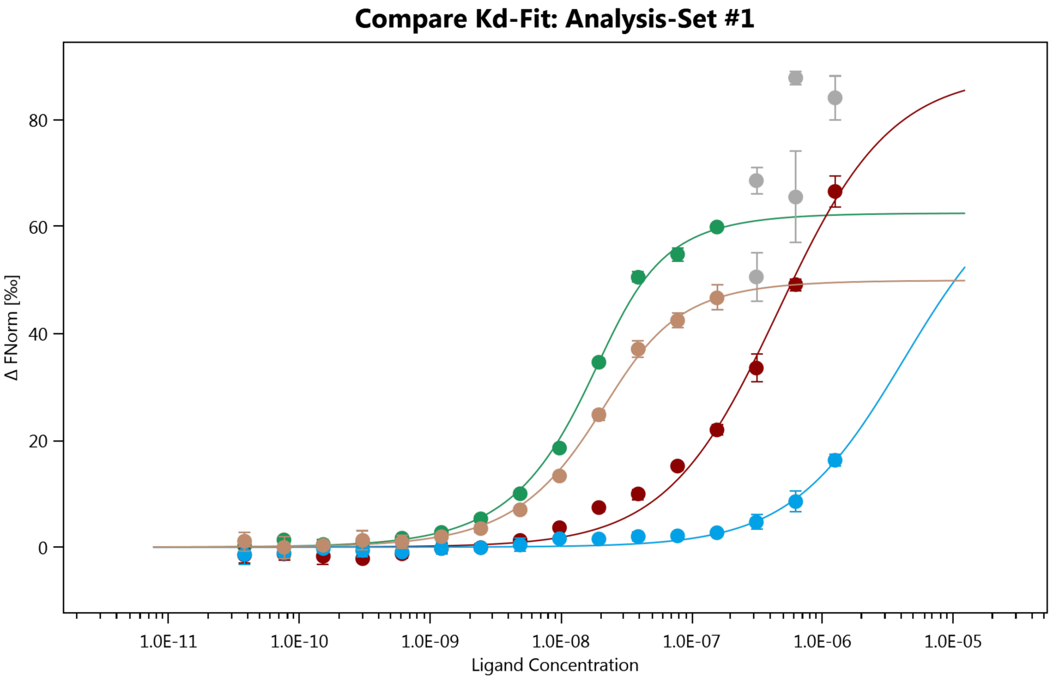

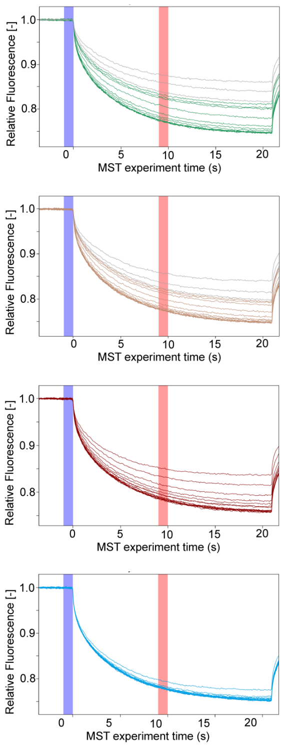

Dataset Overview

Name:	UPF Tray	UPF Tray	UPF Tray	UPF Tray
Graph Color:	•	•	•	•
Target Name:	T35	C35	A35	DNA35
Target Concentration:	20 nM	20 nM	20 nM	20 nM
Ligand Name:	UPF	UPF	UPF	UPF
Ligand Concentration:	156 nM to 0.0381 nM	1.25E+03 nM to 0.0381 nM	1.25E+03 nM to 0.0381 nM	156 nM to 0.0381 nM
n:	3	3	3	3
Comments:				
Excitation Power:	20%	20%	20%	20%
MST Power:	40%	40%	40%	40%
Temperature:	22.0°C	22.0°C	22.0°C	22.0°C
Kd:	6.8186E-09	4.5231E-07	4.2008E-06	9.7778E-09
Kd Confidence:	± 8.5821E-10	± 7.1009E-08	± 3.7335E-06	± 1.0356E-09
Response Amplitude:	62.466699	88.589586	70.296407	49.881493
TargetConc:	2E-08[Fixed]	2E-08[Fixed]	2E-08[Fixed]	2E-08[Fixed]
Unbound:	775.03	787.73	780.27	776.31
Bound:	837.49	876.32	850.57	826.19
Std. Error of Regression:	1.0648309	2.1515979	0.9181774	0.76031158
Reduced χ²:	2.7467454	8.4846222	2.7726497	1.0722463
Signal to Noise:	64.262637	44.227819	82.239504	71.86848

Supplementary Figure S9. hUPF1 interactions with Alexa 647 labeled d(A)$_{35}$, d(C)$_{35}$, d(T)$_{35}$ and DNA$_{35}$ analysed by MST. Reactions were performed as described in materials and methods, main manuscript (*n=3* measurements). A graph of Baseline Corrected Normalized Fluorescence [$^{0}/_{00}$] plotted against hUPF1 concentration (0.0381-1250 nM), along with sample MST traces. Curve fitting was performed using the automated algorithm in the MO.Affinity Analysis software (d(T)$_{35}$ and DNA$_{35}$ 0.0381-156 nM hUPF1; d(A)$_{35}$ and d(C)$_{35}$ 0.0381-1250 nM hUPF1). MST traces were analysed at an MST on time of 10 seconds.

Supplementary Figure S10. hUPF1 unwinding of partially single- and double-stranded DNA helicase substrates with 35 base 5′ homopolymeric tails.

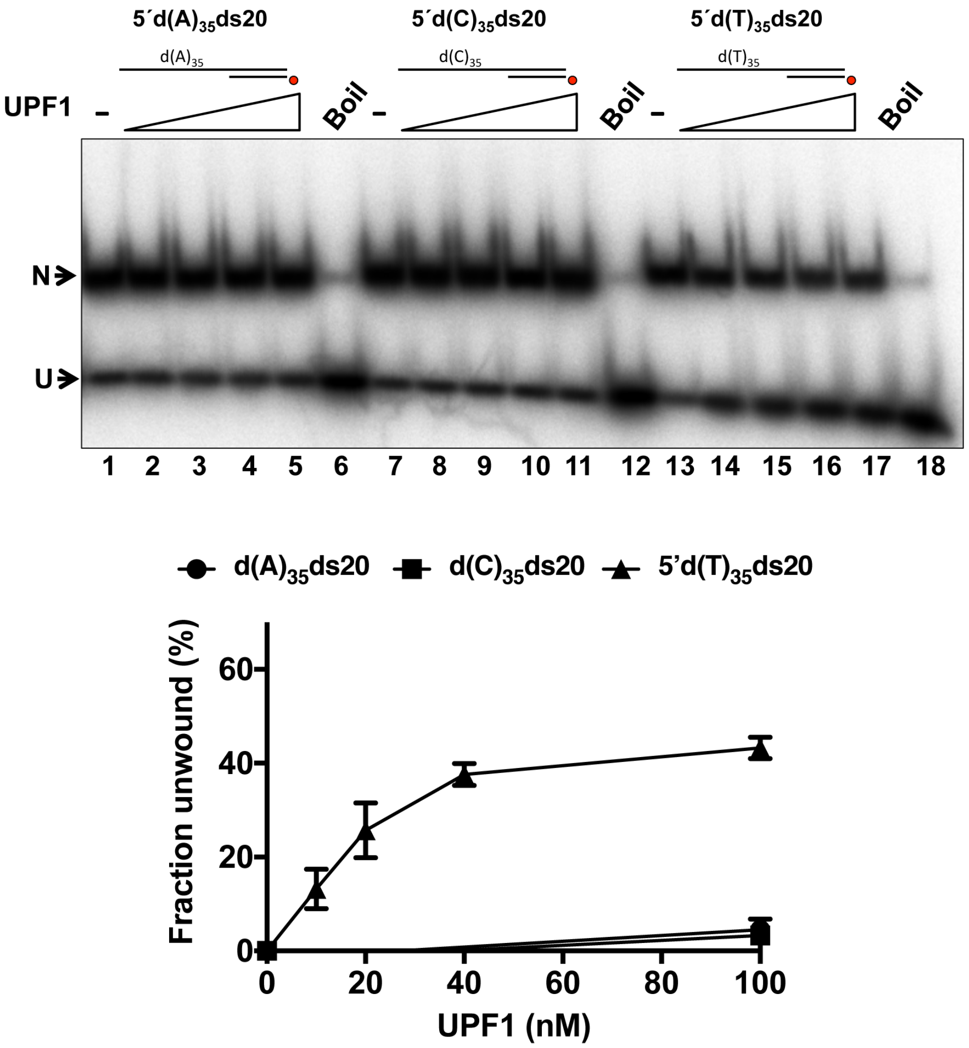

Supplementary Figure S10. DNA helicase substrates with 35 base homopolymeric tails 5′d $(A)_{35}$ and $d(C)_{35}$ were poorly unwound by hUPF1 compared to substrate 5′$d(T)_{35}$ds20. Unwinding reactions were performed under standard conditions (0.2 nM substrate, 10, 20, 40 and 100 nM hUPF1). The graph shows the quantified results from n=4 experimental repeats, mean and standard deviation.

Supplementary Figure S11. hUPF1 fails to bind a DNA triplex motif.

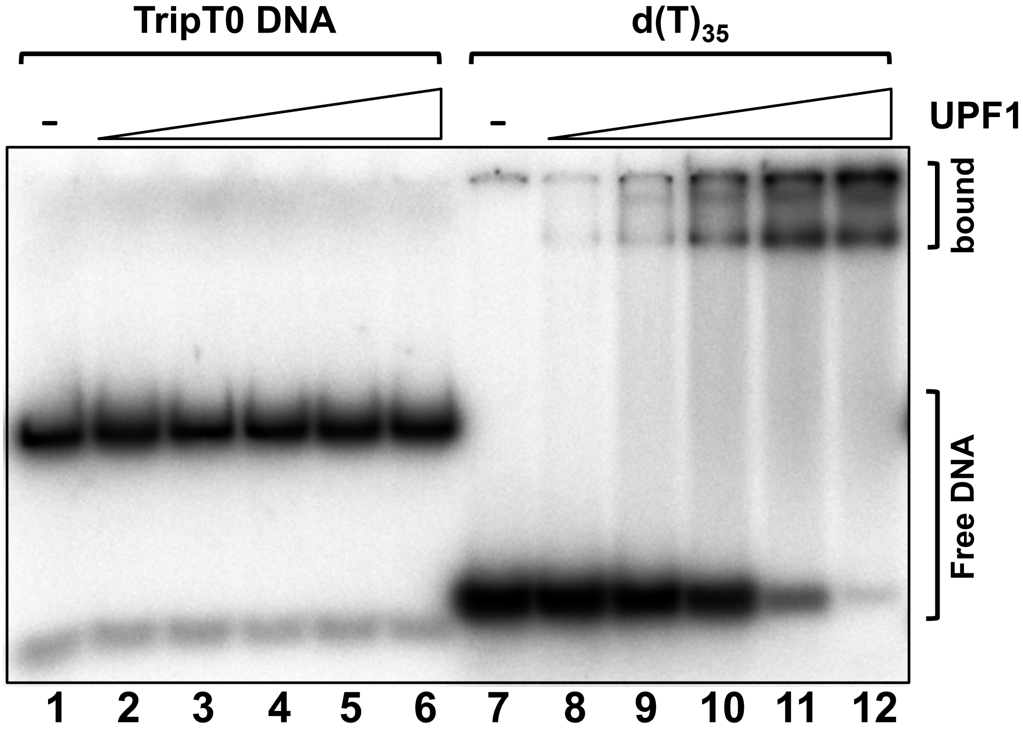

Supplementary Figure S11. hUPF1 fails to bind a DNA triplex motif. Reactions (0.1 nM substrate TripT0 and $d(T)_{35}$, 0.1, 0.5, 2.5, 10 and 40 nM hUPF1) were performed under standard conditions except the reaction, gel and running buffer were supplemented with 10 mM $MgCl_2$ which is required for stability of the triplex substrate. To make substrate TripT0 the 21 base triplex forming oligonucleotide was ^{32}P end-labeled and annealed after heat denaturation of the duplex. No binding was observed to substrate TripT0 or the minor fraction of free 21 base triplex forming oligonucleotide (fast migrating species, lanes 1-6). Binding to control substrate $d(T)_{35}$ (lanes 7-12) demonstrated a propensity for aggregation and retention in the wells in the presence of high $MgCl_2$ concentration (gel and running buffer).
